# Physical-Surface Localization of Aircraft Fuselage Corrosion Using Camera-Calibrated Vision Measurement and Cross-Validated Detector-Center Correction

**DOI:** 10.3390/s26165175

**Published:** 2026-08-15

**Authors:** Chuankun Fang, Changhuan Wang, Zeqing Yang, Kai Peng, Kangni Xu, Jiangpeng Wu, Libin Zhao, Ning Hu

**Affiliations:** 1 School of Mechanical Engineering, Hebei University of Technology, Tianjin 300401, China; chuankunfang628@163.com (C.F.); pengkai@hebut.edu.cn (K.P.); 16622905886@163.com (K.X.); 13898825763@139.com (J.W.); lbzhao@buaa.edu.cn (L.Z.);; 2 Shanghai Institute of Aerospace Systems Engineering, Shanghai 201109, China; wchh007@163.com

**Keywords:** aircraft fuselage corrosion, vision measurement, camera calibration, physical-surface localization, detector-output boundary uncertainty, corrosion inspection, deep-learning-aided perception

## Abstract

Aircraft fuselage corrosion inspection requires both image-domain recognition and metric physical-surface localization for maintenance execution. This study develops a camera-calibrated vision measurement framework that combines PWDE-YOLOv8n-based corrosion perception, original-image coordinate restoration, lens-distortion compensation, ray-based surface mapping, and detector-center bias correction. The perception dataset comprised 2143 images and 5941 corrosion annotations and was partitioned at the physical-specimen, acquisition-session, or source-group level into 1500 training images, 429 validation images, and 214 independent detector-test images. Detailed physical localization was evaluated on a six-image metrology cohort acquired in six sessions, containing 21 corrosion boxes and 84 axial coordinates. A six-fold leave-one-image-out procedure was adopted; in each fold, the center-shift parameters were estimated from the other five images and applied unchanged to the held-out image. The proposed method achieved a mean absolute axial error of 0.641 mm (95% image-cluster bootstrap confidence interval: 0.571–0.708 mm), an RMSE of 0.809 mm, a maximum error of 3.262 mm, and a projected physical-plane bounding-box IoU of 87.12%. The expanded uncertainty of the manually established reference coordinates was 0.374 mm at k = 2, and Monte Carlo propagation produced a mean absolute error of 0.656 mm with a 95% interval of 0.618–0.693 mm. The proposed method reduced the MAE by 97.91% relative to local pixel-to-millimeter scaling and by 70.58% relative to conventional calibrated camera mapping, while producing accuracy comparable to planar homography mapping. Within ρ ≥ 1500 mm, W ≤ 150 mm, and θ ≤ 20°, the estimated curvature-induced additional axial error did not exceed 0.683 mm. A separate ten-image deployment evaluation produced a mean axial error of 2.448 mm and an average processing time of 53.35 ms/image.

## 1. Introduction

The aircraft fuselage is exposed for prolonged periods to high-temperature, high-humidity, and salt-fog environments, making it susceptible to localized metal corrosion such as pitting corrosion and crevice corrosion, which can seriously threaten flight safety [1,2]. If corrosion is not detected in a timely manner and its location is not quantified accurately, structural integrity may be compromised and severe failures may occur [3,4,5,6]. Accurate localization can support targeted maintenance actions, reduce inspection and repair costs, and provide reliable inputs for corrosion assessment and maintenance planning, thereby improving maintenance efficiency and safety assurance [7,8].

Vision-based localization has become an important solution for defect detection and positioning in industrial inspection. Existing methods for defect localization can be broadly categorized into three levels: (i) image-domain localization, (ii) physical-surface localization, and (iii) three-dimensional (3D) coordinate localization.

(i) Image-domain localization estimates target positions in the image coordinate system using pixel-level cues (e.g., edges, grayscale/texture patterns) or high-level semantic features learned by deep networks. Representative studies combine deep learning with imaging geometry to enable automated defect detection and longitudinal localization in constrained scenarios [9]. Data augmentation strategies, including generative approaches, have been used to alleviate class imbalance and improve robustness [10]. Monocular vision-based solutions have also been reported to achieve millimeter-level localization while maintaining real-time performance in maintenance-oriented settings [11]. Extensions to non-conventional imaging configurations (e.g., multi-aperture infrared systems) further exploit calibrated geometric constraints to enhance positioning accuracy under limited visual information [12]. However, image-domain localization remains sensitive to image quality and often degrades under weak texture, low contrast, or non-uniform illumination. Robust visual analysis under anomalous image conditions has also been investigated through directional signal processing and multimodal feature integration. For example, Nuanmeesri and Poomhiran developed an anomalous-image analysis framework that combines Hessian-based ridge detection, gradient-weighted radial voting, multidirectional region-of-interest extraction, signal smoothing, peak detection, and statistical rejection of inconsistent directional observations [13]. Although their application concerns tree-ring analysis rather than aircraft corrosion, the study illustrates a general strategy for improving robustness when local structures are interrupted by cracks, contamination, weak contrast, or other image anomalies. Its multidirectional analysis also shows that relying on a single image direction may overlook off-axis or locally corrupted structural information.

Geometric data augmentation and multimodal fusion provide two additional routes for improving image-domain robustness. Image rotation can increase orientation diversity during detector training; however, arbitrary-angle rotation may crop peripheral content, create artificial boundary pixels, or alter the geometry of a bounding-box annotation if the image and annotation are not transformed consistently [14]. Therefore, rotation augmentation cannot by itself compensate for true out-of-plane viewpoint changes or errors in camera extrinsic parameters. Multimodal fusion methods combining visible images with complementary spectral signals and parallel attention-based feature extraction have demonstrated the potential of complementary sensing for visually ambiguous classification tasks [15]. Beyond industrial inspection, semantically enhanced selective image encryption has used deep salient-object detection to identify semantically sensitive regions of interest before selective encryption, demonstrating that reliable object localization can also support information-security workflows [16]. This application is methodologically distinct from corrosion inspection, but it illustrates the broader role of localization models in region-specific information processing.

(ii) Physical-surface localization maps image coordinates to a known surface coordinate system to obtain defect positions on the physical structure. Coarse localization typically establishes approximate correspondences between image features and surface coordinates, offering high efficiency with reduced manual intervention [17,18,19]. More accurate localization relies on camera calibration and geometric transformations to construct precise image-to-surface mappings, and has been widely applied in high-precision industrial positioning tasks such as weld seam and rivet localization [20,21]. Nevertheless, performance can still degrade in adverse lighting or weak-texture regions, and computational burden may increase in large-scale inspections.

(iii) 3D coordinate localization reconstructs defect positions in three-dimensional space using stereo vision or depth-sensing modalities. By calibrating intrinsic/extrinsic parameters and converting image observations into world coordinates, such methods provide full spatial characterization and have achieved high accuracy in representative applications [22,23,24]. However, 3D localization typically requires additional sensing or computation and may be challenging to deploy for near-real-time, large-area field inspection.

However, existing studies are often evaluated using different datasets, imaging configurations, and error definitions, making direct comparison difficult. In particular, quantitative comparisons among simple pixel-scale conversion, planar homography, and camera-calibrated physical-surface mapping under the same detector outputs and physical-coordinate references remain limited. Therefore, a same-platform comparison under a leakage-controlled cross-validated physical-localization protocol is required to clarify the practical contribution of the proposed framework.

Motivated by these limitations, this work targets reliable corrosion detection and quantitative physical localization on aircraft fuselage surfaces using a calibrated monocular vision system, aiming to provide accurate physical localization under the current calibrated setup and on locally planar fuselage regions. Although vision-based defect inspection has advanced considerably, most existing studies still focus on image-space recognition, where the output is limited to pixel-level bounding boxes or masks. For aircraft fuselage maintenance, however, identifying a corrosion region in the image is not sufficient. Maintenance personnel also require its corresponding physical location on the fuselage surface to support targeted inspection, area-based assessment, and repair execution. In addition, existing studies are typically evaluated mainly by detection metrics such as mAP, while maintenance-oriented indicators, including physical localization accuracy and end-to-end runtime, are much less frequently assessed in a unified framework.

In detector-based corrosion perception, it is necessary to distinguish image-formation edge spreading from detector-output bounding-box bias. During image acquisition, the ideal surface radiance distribution is blurred by the optical point-spread function and discretized by sensor sampling, which converts an ideal sharp boundary into a finite-width intensity transition. This image-formation effect affects the observability of the corrosion boundary but does not necessarily produce a systematic displacement of the boundary location. Detector-output bounding-box bias is a downstream phenomenon defined by the coordinate residual between a predicted box and its manually annotated reference box. In addition to image-formation blur, it may be affected by image resizing, feature-map downsampling, receptive-field aggregation, multi-scale feature fusion, bounding-box regression, non-maximum suppression, weak boundary contrast, and annotation ambiguity. Therefore, image-formation edge spreading is treated as one possible upstream contributor to detector-boundary uncertainty rather than as being mathematically equivalent to bounding-box bias. When detector-generated box coordinates are directly projected onto the fuselage surface, the resulting center displacement or size deviation is transferred into millimeter-scale physical-coordinate error. Therefore, the present study estimates and corrects the measurable detector-output center bias rather than assuming a universal convolution-induced broadening coefficient.

Existing defect-detection studies often stop at image-space localization, whereas our work further maps detected corrosion regions to physical-surface coordinates for maintenance-oriented positioning. Considering the practical requirements of aircraft fuselage inspection, this study develops an integrated monocular vision framework that combines corrosion detection, camera calibration, image-to-surface coordinate mapping, and physical localization evaluation. Under the current calibrated operating setup, the method aims to provide accurate corrosion localization on locally planar fuselage regions while maintaining efficient inspection speed.

To this end, a camera-calibrated detection-to-surface localization framework for aircraft fuselage surfaces is established in this study. The main contributions are summarized as follows:

(1) Camera-calibrated vision measurement framework: A vision-based measurement framework is developed for aircraft fuselage corrosion localization, integrating deep-learning-aided corrosion perception with calibrated physical-surface coordinate mapping.

(2) Leakage-controlled detector-center correction: A six-fold leave-one-image-out procedure is introduced to estimate detector-center bias without allowing a held-out image to contribute to its own correction parameters.

(3) Camera-calibrated detection-to-surface conversion: Detector-generated boxes are restored to the original-image space, corrected for lens distortion, mapped onto the local physical surface, and compensated using the fold-specific center shift.

(4) Maintenance-oriented quantitative evaluation: Physical localization is evaluated using axial error, projected physical-plane IoU, image-cluster bootstrap confidence intervals, reference-measurement uncertainty propagation, same-platform baselines, and a quantified curvature-applicability range.

## 2. Materials and Methods

### 2.1. Aircraft Fuselage Surface Corrosion Types and Localization Requirements

**Figure 1:**
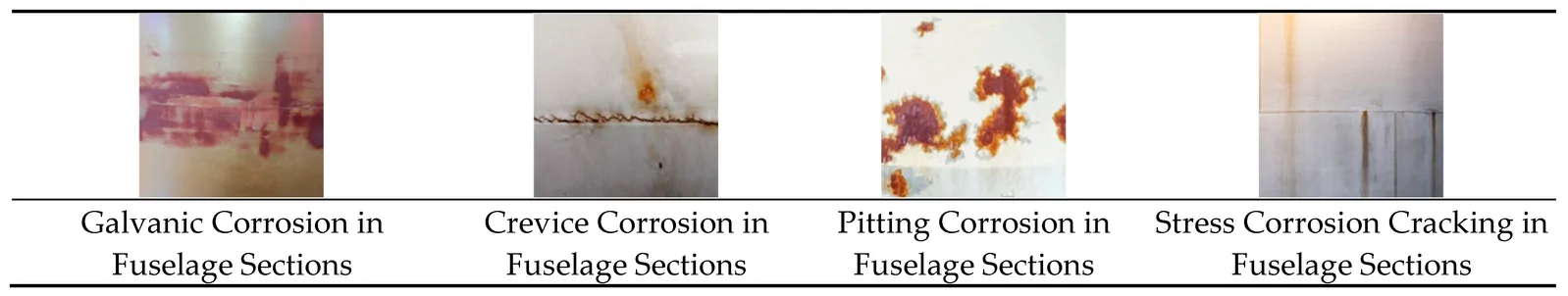
Representative corrosion images on aircraft fuselage surfaces.

Common forms of corrosion on aircraft fuselage skin surfaces—particularly around fasteners [25,26] and at skin-to-frame junctions—include galvanic corrosion, crevice corrosion, pitting corrosion, and stress corrosion cracking, as illustrated in Figure 1 [27]. These corrosion types differ considerably in morphology and formation mechanism. In practical inspection, corrosion regions may exhibit small affected areas, weak texture, complex structural backgrounds, and low contrast relative to the surrounding surface, which increases the difficulty of reliable identification and localization. Accordingly, improving the accuracy and speed of corrosion identification and localization on fuselage surfaces has become a key research focus. This study focuses on pitting corrosion because it is a representative form of localized corrosion and frequently exhibits small affected areas, weak texture, and low contrast, which make automated inspection particularly challenging [28,29]. Pitting corrosion typically appears as small and irregular surface depressions and may accelerate local material degradation. Under pressurization and cyclic loading, corrosion pits can act as stress concentrators, thereby increasing the risk of crack initiation and structural damage [30,31].

Identifying a corrosion region in the image domain alone is insufficient for aircraft maintenance. In addition to corrosion recognition, maintenance-oriented inspection also requires the corresponding physical-surface location of the detected corrosion region so that subsequent measurement, assessment, and repair can be carried out. Therefore, this study addresses both corrosion recognition and physical localization on the local fuselage surface plane.

### 2.2. Aircraft Fuselage Surface Corrosion Identification and Localization

**Figure 2:**
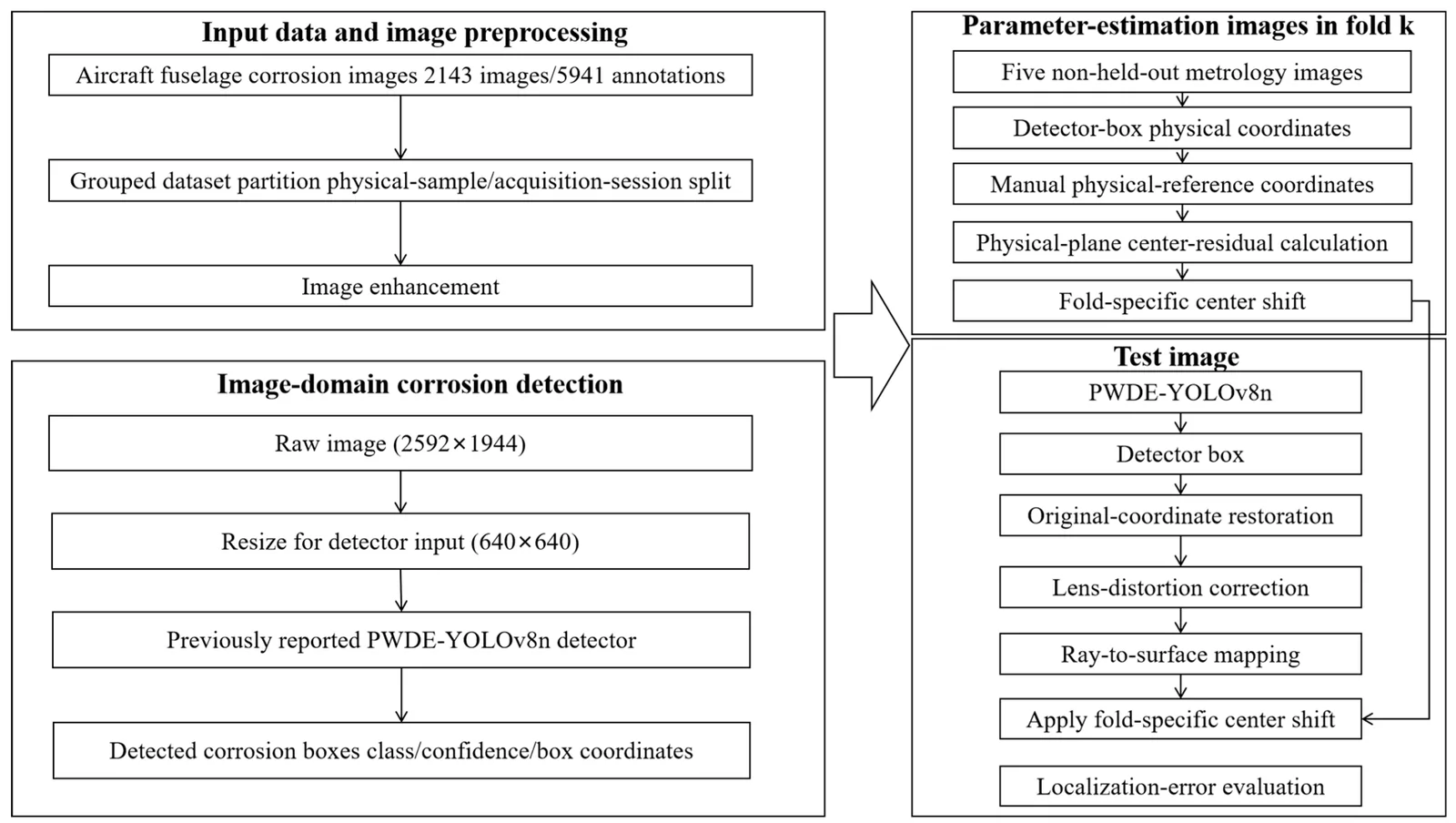
Overall framework of the proposed detection-to-surface corrosion localization pipeline with Cross-Validated Detector-Center Correction.

A camera-calibrated corrosion localization framework is developed for aircraft fuselage surfaces by combining the PWDE-YOLOv8n-based corrosion perception module with a calibrated camera-based coordinate mapping model. The framework consists of five main stages: dataset preparation, image preprocessing, detector-based corrosion perception, Cross-Validated Detector-Center Correction, and physical-surface localization. First, corrosion images provide image-domain observations and annotations for corrosion perception. Second, image enhancement and data augmentation are applied to improve image quality and sample diversity. Third, PWDE-YOLOv8n identifies corrosion regions in the image domain. The raw images were acquired at a resolution of 2592 × 1944 and directly resized to 640 × 640 for detector training and inference. After inference, the detector-generated corner coordinates were restored from the detector-input space to the original-image coordinate system. The restored coordinates were then corrected using the calibrated lens-distortion parameters and mapped onto the local fuselage surface plane through ray-to-surface intersection. After physical-surface mapping, the fold-specific physical-plane center shift was subtracted from both mapped corners. The overall framework is shown in Figure 2.

### 2.3. Dataset Composition, Leakage-Free Partitioning, and Corrosion Perception Module

The corrosion images used in this study were derived from a previously established aircraft fuselage surface corrosion dataset. The dataset provides image-domain corrosion observations and bounding-box annotations for training the corrosion detector and evaluating the subsequent physical-surface localization framework.

The dataset consists of 2143 original images and 5941 corrosion annotations collected from three complementary sources: laboratory-prepared simulated corrosion samples, fuselage-related experimental corrosion samples, and screened real-scene corrosion images. The simulated corrosion samples and screened real-scene images were prepared or collected with reference to standard corrosion exposure and web-screening procedures [32,33,34]. These sources were included to increase the diversity of corrosion morphology, surface background, defect scale, and illumination condition. In the current dataset inventory, the fuselage-related experimental samples and screened real-scene images are jointly represented by the “Real corrosion” class, which contains 594 images and 1430 annotations. The role of each source in the present study is summarized in Table 1.

To prevent information leakage, dataset partitioning was performed using group-level identifiers rather than random image-level splitting. For laboratory-prepared and fuselage-related experimental images, each image was assigned a physical-specimen identifier and an acquisition-session identifier. All repeated views, different camera poses, local crops, and multiple acquisition sessions originating from the same physical specimen were assigned to one and only one subset. When several acquisition sessions were associated with the same specimen, the physical-specimen identifier took precedence.

Six calibrated images within the independent detector-test set were selected to form the detailed physical-localization metrology cohort. All images associated with the corresponding specimens or acquisition sessions were excluded from detector training and model selection. Within the metrology cohort, each held-out image was excluded from the estimation of its own fold-specific center-shift parameters.

Data augmentation was performed only after the group-level partitioning had been completed and was applied exclusively to the training subset. For geometric augmentation, each transformed image and its bounding-box annotations were updated using the same transformation matrix. Rotation augmentation was used only for detector training and was not applied to the validation images, the independent detector-test set, or the six-image metrology cohort. Rotation augmentation was used only to improve image-domain orientation robustness during detector training.

Because some candidate localization images had participated in the original detector training process, the dataset was repartitioned and PWDE-YOLOv8n was retrained from the original pretrained initialization. The previous detector checkpoint was not reused. The network architecture, input resolution, optimizer, learning-rate schedule, training epochs, and non-maximum-suppression settings were kept consistent with the original experimental configuration. Model selection and confidence-threshold determination were performed using only the validation subset.

The revised dataset contained 1500 training images from 18 specimen/session or source groups, 429 validation images from 6 groups, and 214 independent detector-test images from 6 groups, containing 4159, 1189, and 593 corrosion annotations, respectively. The 214-image detector-test set was used exclusively for final image-domain detector evaluation. Within this set, six calibrated images acquired in six sessions and containing 21 corrosion boxes formed the detailed physical-localization metrology cohort. Because the center-shift parameters for each evaluated image were estimated from the other five cohort images, the physical-localization results constitute six-fold leave-one-image-out cross-validated evidence rather than an independent external metrology test.

The 214-image independent detector-test set was used to assess image-domain corrosion-perception performance. Within this set, six calibrated images acquired in six sessions and containing 21 corrosion boxes formed the detailed physical-localization metrology cohort; their cross-validated localization results were reported separately from the 214-image detector metrics. The additional ten-image online deployment evaluation was treated as a separate engineering replication.

As shown in Table 2, the independent detector-test groups and their associated images were completely separated from detector training and model selection. A dataset-manifest audit was conducted after partitioning to verify that the intersections of the specimen, acquisition-session, source-group, and parent-image identifiers among the three subsets were empty.

The validation subset was used only for detector model selection and confidence-threshold determination. It was not used to fit the physical-coordinate center-shift parameters reported for the six-image metrology cohort. Detector-center correction within that cohort was evaluated using the leave-one-image-out procedure described in Section 2.5.2.

For image-domain corrosion extraction, PWDE-YOLOv8n was adopted as the corrosion perception module. The detector was previously reported as a lightweight corrosion detector [35]. In the present study, it is used to generate corrosion classes, confidence scores, and bounding-box coordinates, while the methodological focus is the conversion of detector-generated boxes into calibrated physical-surface coordinates. Therefore, the detector output is treated as a perception result with possible boundary uncertainty rather than as a direct physical measurement of the corrosion boundary.

During inference, the original images acquired at 2592 × 1944 pixels were resized to 640 × 640 pixels for detector input. After non-maximum suppression, the retained bounding boxes were restored from the detector-input coordinate system to the original-image coordinate system. The restored coordinates were subsequently corrected using the calibrated lens-distortion parameters and mapped onto the local physical surface through the camera-calibration model. The detailed detector architecture and module-level ablation results have been reported in [26] and are therefore not repeated here.

### 2.4. Six-Image Cross-Validated Metrology Cohort and Physical-Reference Construction

The six-image physical-localization metrology cohort was fixed before the cross-validated evaluation. For each corrosion region, the detector-generated upper-left and lower-right bounding-box corners were restored from the 640 × 640 detector-input space to the original-image coordinate system, corrected for lens distortion, and mapped onto the local physical-surface plane using the calibrated camera model.

The physical reference for each corrosion region was defined as the axis-aligned minimum bounding rectangle enclosing the visible corrosion area on the local surface plane. The coordinate origin and surface axes were established using the same calibration-board reference and origin-transfer procedure adopted in the camera-mapping experiment. The upper-left and lower-right reference coordinates were independently measured in the surface X- and Y-directions.

Each reference coordinate was independently measured three times by each of two operators, yielding six measurements per coordinate (M = 6). The arithmetic mean of the six measurements was used as the final physical-coordinate reference.

#### Physical-Reference Measurement Uncertainty and Propagation

The manually measured physical coordinates were treated as reference values with finite measurement uncertainty rather than as error-free ground truth. For each corrosion region, the upper-left and lower-right boundary coordinates along the local surface X- and Y-directions were independently marked and measured by two operators. Each operator repeated the complete marking and measurement procedure three times in randomized order. The localization results generated by the proposed software were not displayed during reference construction.

For the *i*-th physical reference coordinate, the final reference value was calculated as

(1) $$q_{i}^{ref}=\frac{1}{M}\sum_{m=1}^{M}q_{i,m}$$

where M = 6 denotes the total number of measurements obtained from two operators with three repetitions each.

The standard deviation of the repeated measurements was calculated as

(2) $$s_{req,i}=\sqrt{\frac{1}{M-1}\sum_{m=1}^{M}{\left(q_{i,m}-q_{i}^{ref}\right)}^{2}}$$

The Type-A standard uncertainty associated with the averaged reference coordinate was then estimated as

(3) $$u_{rep,i}=\frac{s_{rep,i}}{\sqrt{M}}$$

The engineering measurement tape had a resolution of *d* = 0.1 mm. Assuming that the rounding error was uniformly distributed within one resolution interval, the corresponding standard uncertainty was

(4) $$u_{res}=\frac{d}{\sqrt{12}}$$

Measurement uncertainty was propagated to the reported physical-localization metrics using 10,000 Monte Carlo trials. In each trial, the manually measured reference coordinates were randomly perturbed according to their estimated uncertainty components. Coordinate-system establishment uncertainty was modeled as a common displacement shared by all reference coordinates belonging to the same image, whereas repeated marking and coordinate-reading uncertainties were modeled as coordinate-specific random components.

During each Monte Carlo trial, the predicted physical coordinates were kept unchanged, while the sampled reference coordinates were used to recalculate the axial localization errors, RMSE, and projected bounding-box IoU. The median and the 2.5th–97.5th percentile range of the resulting metric distributions were reported as the measurement-uncertainty-propagated results.

The uncertainty associated with establishing and transferring the local surface-coordinate origin and axes was evaluated through repeated coordinate-system reconstruction experiments. The resulting standard deviation was denoted by *u*_setup_. When available, the calibrated gauge-block uncertainty *u*_gb_ was obtained from its calibration certificate.

Assuming that the individual uncertainty components were mutually independent, the combined standard uncertainty of the *i*-th reference coordinate was calculated as

(5) $$u_{ref,i}=\sqrt{u_{rep,i}^{2}+u_{res}^{2}+u_{setup}^{2}+u_{gb}^{2}}$$

The corresponding expanded uncertainty was reported as

(6) $$U_{ref,i}=ku_{ref,i}$$

where a coverage factor of *k* = 2 was used to provide an approximate 95% coverage interval.

### 2.5. Image-Formation Edge Spreading and Cross-Validated Detector-Center Correction

#### 2.5.1. Image-Formation Edge Spreading Versus Detector-Output Box Bias

To distinguish the image-signal formation mechanism from detector-stage localization error, the observed image is modeled as

(7) I(x,y)=[O(x,y)∗h(x,y)]↓s+n(x,y)

where *O*(*x*,*y*) denotes the ideal projected radiance distribution of the inspected surface, *h*(*x*,*y*) denotes the optical point-spread function, ∗ denotes convolution, ↓s represents discrete sensor sampling, and *n*(*x*,*y*) denotes imaging noise.

For theoretical illustration, a one-dimensional ideal boundary may be represented as a step edge:

(8) $$O(x)\;=\;A_{0}\;+\;(A_{1}-A_{0})H(x\;-\;x_{0})$$

where *x*_0_ denotes the ideal boundary location and H(⋅) is the Heaviside step function. When the point-spread function is approximated by a Gaussian function,

(9) $$h_{\sigma }(x)=\frac{1}{\sqrt{2\pi }\;\sigma }exp(-\frac{x^{2}}{2\sigma^{2}})$$

the normalized edge-spread response becomes

(10) $$E(x)=\frac{1}{2}[1+erf(\frac{x-x_{0}}{\sqrt{2}\sigma })]$$

The edge-spread response above shows that optical convolution converts an ideal discontinuous boundary into a finite-width intensity transition.

(11) $$w_{10–90}=2\sqrt{2}\sigma {erf}^{-1}(0.8)\approx 2.563\sigma$$

The quantity *w*_10–90_ characterizes image-domain edge spreading and should not be interpreted directly as detector bounding-box displacement. For a symmetric point-spread function, the 50% intensity location remains at *x*_0_; therefore, symmetric optical blurring alone broadens the transition region but does not necessarily create systematic boundary translation or box expansion.

If a boundary-extraction process responds to an intensity level *T*, its apparent edge location can be written as

(12) $$x_{T}=x_{0}+\sqrt{2}\sigma {erf}^{-1}(2T-1)$$

Thus, apparent boundary displacement may occur when the effective response level differs from 0.5 or when the imaging response is affected by asymmetric illumination, surface reflection, noise, sampling, or local contrast variation. In a deep detector, these image-domain effects are further transformed by feature-map downsampling, receptive-field aggregation, multi-scale feature fusion, bounding-box regression, and post-processing.

Accordingly, detector-output box bias is defined separately as the residual between the predicted box

(13) $$B_{i}^{p}=(u_{1,i}^{p},v_{1,i}^{p},u_{2,i}^{p},v_{2,i}^{p})$$

and its manually annotated reference

(14) $$B_{i}^{g}=(u_{1,i}^{g},v_{1,i}^{g},u_{2,i}^{g},v_{2,i}^{g})$$

Image-formation edge spreading and detector-output box bias therefore differ in both processing stage and mathematical description. Edge spreading occurs before detector inference and is characterized by an edge-spread width such as *w*_10–90_. Detector-output box bias occurs after feature extraction, box regression, and post-processing and is characterized by coordinate, center, and size residuals between $B_{i}^{p}$ and $B_{i}^{g}$. In the present study, optical edge spreading is regarded as one possible contributor to the observable boundary uncertainty, whereas the six-fold cross-validated physical-plane center-shift procedure directly estimates and corrects the measurable detector-output box bias.

Table 3 shows that image-formation edge spreading is not treated as a synonym for detector-output box bias. The former explains one mechanism affecting boundary observability, while the latter is the measurable quantity directly used by the proposed correction module.

#### 2.5.2. Cross-Validated Detector-Center Correction

Detector-output box deviations may contain both center displacement and size deviation. The ablation results showed that center-shift correction alone produced the best overall physical-localization performance; therefore, it was retained as the final correction strategy.

Because physical-reference coordinates were available for only six detailed localization images, a six-fold leave-one-image-out procedure was adopted. In fold *k*, image *k* was held out, and the center-shift parameters were estimated using only the corrosion boxes from the other five images. No annotation, physical-coordinate error, or localization result from the held-out image was used to determine its correction parameters.

For a detector-generated box mapped onto the physical surface, let its raw corner coordinates be

(15) $$B_{i}^{raw}=(X_{1,i}^{raw},Y_{1,i}^{raw},X_{2,i}^{raw},Y_{2,i}^{raw})$$

The fold-specific center-bias vector was denoted as

(16) $$\Delta^{\left(k\right)}={\left[\Delta X^{\left(k\right)},\Delta Y^{\left(k\right)}\right]}^{T}$$

The corrected coordinates were calculated as

(17) $$X_{q,i}^{corr}=X_{q,i}^{raw}-\Delta X^{\left(k\right)},\;Y_{q,i}^{corr}=Y_{q,i}^{raw}-\Delta Y^{\left(k\right)},\;q\in \left\{1,2\right\}$$

The same shift was applied to both corners; therefore, the mapped box width and height were preserved. The six held-out folds were pooled only after all fold-specific parameters had been fixed. Consequently, the reported result is an out-of-fold cross-validated estimate rather than a correction fitted directly to the evaluated image.

## 3. Camera Mapping Model

Metal corrosion on aircraft fuselage surfaces can be initially localized in the image domain using a detector-based corrosion perception module. However, accurate corrosion repair requires precise mapping of the detected corrosion regions onto the actual physical surface [36,37]. To address this requirement, a localization method based on a camera mapping model is proposed. The localization framework involves multiple coordinate systems, including the image coordinate system, camera coordinate system, and world coordinate system. Camera intrinsic and extrinsic parameters are first obtained through calibration. Based on these parameters, geometric and projection transformations are applied to establish an accurate mapping between image pixel coordinates and physical-surface coordinates. Through this mapping process, corrosion regions detected in the image plane are transformed into their corresponding positions in physical space. Calibration plate images are used to determine the transformation parameters and ensure accurate coordinate conversion between the involved coordinate systems [38]. This procedure provides reliable spatial information to support subsequent corrosion analysis and precise repair operations.

### 3.1. Establishment of the Camera Mapping System Coordinate System

As the core hardware for the localization of metal corrosion on the aircraft fuselage surface, the camera imaging system projects the fuselage surface and the calibration plate from three-dimensional space onto a two-dimensional imaging plane. To establish an accurate mapping relationship, a series of coordinate systems must be constructed within the camera imaging system. Within the camera mapping framework, metal corrosion observed on the imaging plane is represented in pixel units, whereas corrosion on the aircraft fuselage surface in three-dimensional space is expressed in physical measurement units. Accordingly, two coordinate systems are defined on the imaging plane: the corrosion pixel coordinate system (o-uv) and the image coordinate system (o-xy), which together constitute the initial reference for subsequent coordinate transformations. The origin of the corrosion pixel coordinate system is located at the top-left corner of the image plane, with coordinates measured in pixels. In contrast, the origin of the image coordinate system is defined at the camera’s optical center, whose corresponding location in the corrosion pixel coordinate system is denoted as (u_0_, v_0_).

**Figure 3:**
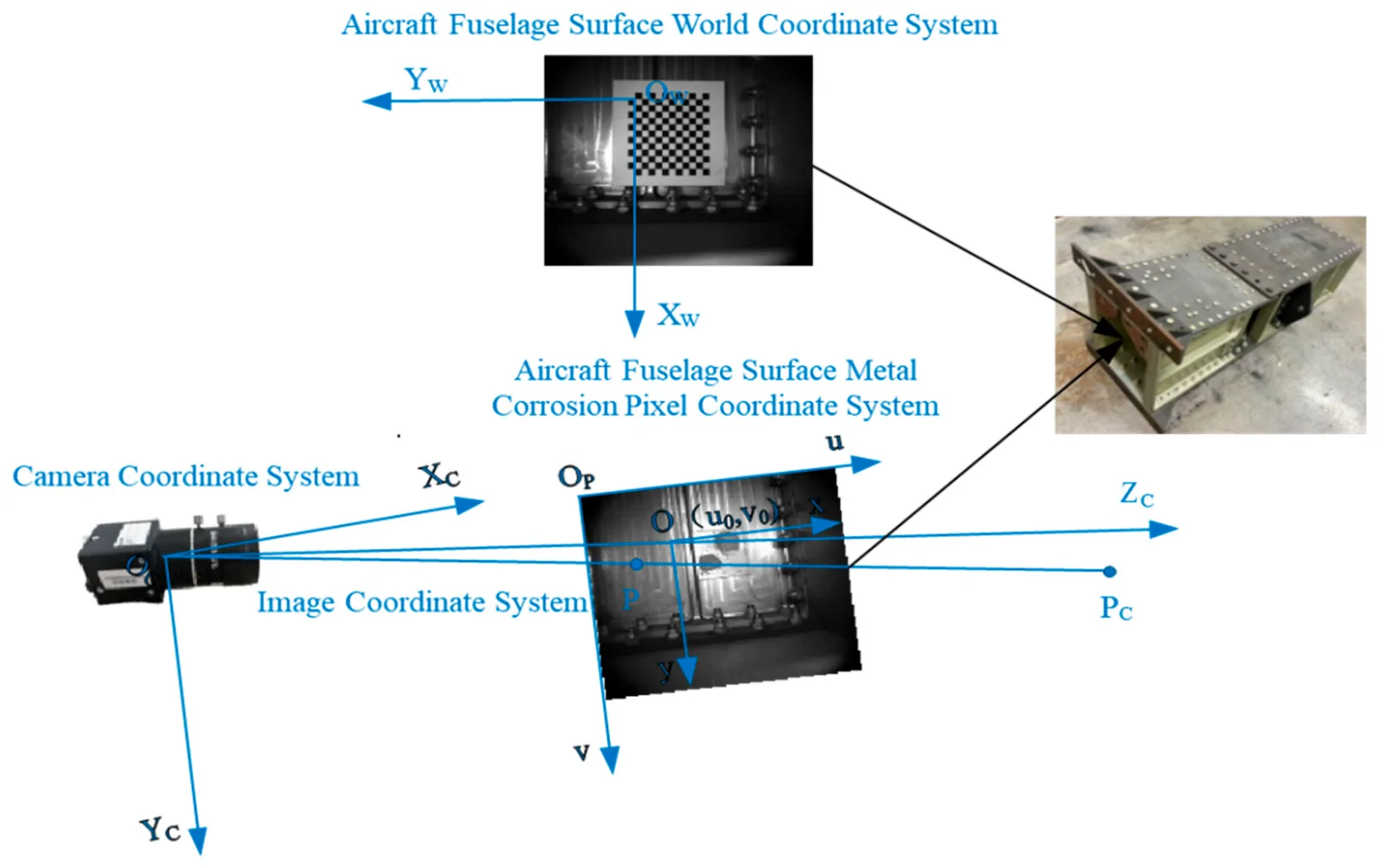
Coordinate systems used in the camera mapping model.

To clarify the positional relationship among the camera, the aircraft fuselage surface, and the calibration plate, both the camera coordinate system and the world coordinate system are defined. The camera coordinate system, denoted as (O_C_-X_C_Y_C_Z_C_), shares the same origin as the image coordinate system, with the X_C_ and Y_C_ axes aligned with the image x-axis and y-axis, respectively. The Z_C_ axis is perpendicular to the imaging plane and points outward from the camera, and all coordinates are expressed in millimeters. The world coordinate system of the aircraft fuselage surface, denoted as (O_W_-X_W_Y_W_ZW), is established to describe the physical space for corrosion localization. The calibration plate serves as the reference plane of the world coordinate system and is defined as the XW-YW plane, with ZW = 0. In the present study, ZW = 0 represents the tangent-plane approximation of a limited local inspection region rather than the geometry of the entire fuselage. Its applicable curvature range is quantitatively defined in Section 3.6. The upper-left inner corner of the calibration plate is selected as the origin of the world coordinate system, as illustrated in Figure 3.

### 3.2. Establishment of the Camera Linear Model

The camera mapping linear model is established under ideal imaging conditions, assuming that lens distortion is absent and that the physical-surface corrosion feature points on the aircraft fuselage, the optical center, and the corresponding image points are collinear. The image captured by the camera represents the mapping and transformation of the physical-surface feature points across different coordinate systems.

**Figure 4:**
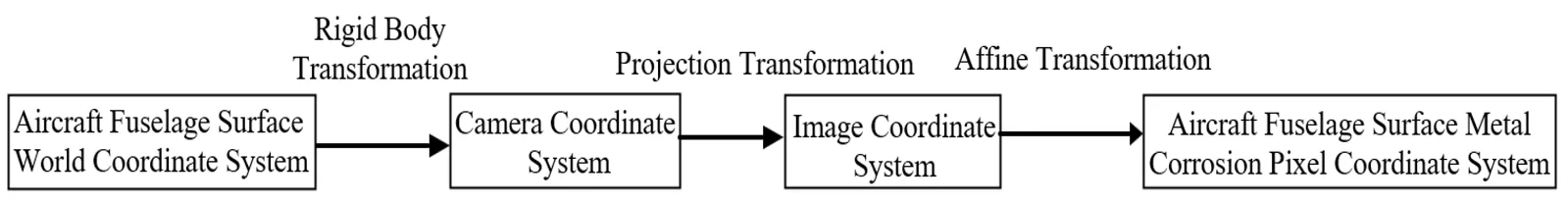
Pixel-to-surface mapping process based on camera calibration.

Through rigid-body transformation, these points are first mapped into the camera coordinate system and then projected into the corrosion pixel coordinate system through the calibrated camera model, as shown in Figure 4.

(1) Transformation from the Aircraft Fuselage Surface World Coordinate System to the Camera Coordinate System

A point located in the aircraft fuselage surface world coordinate system is transformed into the corresponding point in the camera coordinate system through a rigid-body transformation. The transformation relationship between the two coordinate systems is given by

(18) $$\left[\begin{array}{c} X_{c}\\ Y_{c}\\ Z_{c} \end{array}\right]=\;R\left[\begin{array}{c} X_{w}\\ Y_{w}\\ Z_{w} \end{array}\right]\;+\;t$$

where **R** is the rotation matrix and **t** is the translation vector from the aircraft-fuselage surface coordinate system to the camera coordinate system.

(2) Transformation from the Camera Coordinate System to the Image Coordinate System

The transformation from the camera coordinate system to the image coordinate system essentially involves projecting the target from the three-dimensional physical space into the image coordinate system using the camera’s intrinsic parameter matrix as the bridge. The transformation relationship is given by

(19) $$\lambda \left[\begin{array}{c} u\\ v\\ 1 \end{array}\right]=\left[\begin{array}{ccc} fx & \gamma & cx\\ 0 & fy & cy\\ 0 & 0 & 1 \end{array}\right]\left[\begin{array}{cc} R & t \end{array}\right]\left[\begin{array}{c} X_{w}\\ Y_{w}\\ \begin{array}{c} Z_{w}\\ 1 \end{array} \end{array}\right]$$

where λ is the homogeneous scale factor, (u,v) are the pixel coordinates in the image plane, **R** and **t** are the extrinsic rotation matrix and translation vector, and **K** is the intrinsic matrix. Here, fx and fy denote the focal lengths in pixel units along the horizontal and vertical directions, respectively; (cx,cy) denotes the principal point; and γ is the skew parameter.

(3) Establishment of the Linear Model

Therefore, under ideal imaging conditions, the world-to-pixel mapping can be summarized as

(20) $$\lambda p=K[\begin{array}{cc} R & t \end{array}]P_{\omega }$$

where λ is the homogeneous scale factor; $\tilde{p}={\left[u,v,1\right]}^{T}\in R^{3}$ is the homogeneous image point; $\tilde{P_{W}}={\left[X_{W},Y_{W},Z_{W},1\right]}^{T}$ is the homogeneous world point; $K\in R^{3\times 3}$ is the intrinsic matrix; $R\in SO(3)\subset R^{3\times 3}$ is the rotation matrix; and $t\in R^{3\times 1}$ is the translation vector.

(21) $$Z_{C}\left[\begin{array}{c} u\\ v\\ 1 \end{array}\right]=\left[\begin{array}{cccc} f_{x} & 0 & u_{0} & 0\\ 0 & f_{y} & v_{0} & 0\\ 0 & 0 & 1 & 0 \end{array}\right]\left[\begin{array}{cc} R & T\\ 0^{T} & 1 \end{array}\right]\left[\begin{array}{c} X_{W}\\ Y_{W}\\ \begin{array}{c} Z_{W}\\ 1 \end{array} \end{array}\right]$$

The camera’s intrinsic parameters are determined by the hardware system design and are independent of the imaged object. For the same industrial camera, the intrinsic parameter matrix remains consistent across multiple captures. The position and orientation of the aircraft fuselage surface determine the camera’s extrinsic parameters. When the distance between the imaging plane and the camera changes, the translation vector changes accordingly, while the rotation matrix is determined by the direction of the imaging plane. Within the same spatial plane, the coordinate transformation for all points follows the same extrinsic parameters. Therefore, under the premise that neither the aircraft fuselage surface for corrosion recognition nor the industrial camera moves, the intrinsic and extrinsic parameter matrices of the camera remain unchanged.

### 3.3. Establishment of the Camera Nonlinear Model

The linear model describes the mapping relationship of the camera under ideal imaging conditions, which contrasts with the real model in actual imaging processes. During the image acquisition of metal corrosion on the aircraft fuselage surface, the geometric distortion of the camera leads to deviations in the position of the pixels on the imaging plane, affecting the imaging accuracy across the entire field of view. This results in a mismatch between the actual corrosion targets and the ideal camera imaging model, thus causing lens distortion. While the inherent distortion characteristics of the camera lens cannot be fully eliminated, their impact can be reduced through various corrective measures. Based on a quantitative analysis of lens distortion, the camera nonlinear model is derived in conjunction with the linear model. Lens distortion is typically divided into tangential distortion and radial distortion.

(1) Radial Distortion

Radial distortion, denoted as $d_{r\;}$, arises from the distortion caused by changes in the radial curvature of the optical lens, typically due to design flaws in the optical system. For example, manufacturing issues during the lens production process that cause changes in radial curvature can be represented by the following formula:

(22) $$\left\{\begin{array}{c} x^{\prime }\;=\;x\left[1\;+\;k_{1}\left(x^{2}\;+\;y^{2}\right)\;+{\;k}_{2}{\left(x^{2}\;+\;y^{2}\right)}^{2}\right]\\ y^{\prime }\;=\;y\left[1\;+\;k_{1}\left(x^{2}\;+{\;y}^{2}\right)+k_{2}{\left(x^{2\;}+\;y^{2}\right)}^{2}\right] \end{array}\right.$$

where $\left(x^{\prime },y^{\prime }\right)$ are the actual (distorted) image coordinates, $\left(x,y\right)$ are the image coordinates under the ideal camera mapping model. The first-order and second-order radial distortion coefficients are represented by *k*_1_ and *k*_2_, respectively. The vector ***k***, composed of these two coefficients is defined as the distortion vector.

(2) Tangential Distortion

Due to manufacturing and assembly errors during production, the lens plane of the camera cannot remain perfectly parallel to the imaging plane, causing an angular variation between the lens’s geometric center and the optical center. This results in tangential distortion, denoted as $d_{t}$, which can be expressed by the following formula:

(23) $$\left\{\begin{array}{c} x^{\prime }\;=\;x\;+\;2p_{1}xy\;+\;p_{2}\left(3x^{2}\;+\;y^{2}\right)\\ y^{\prime }\;=\;y\;+\;2p_{2}xy\;+{\;p}_{1}\left(r^{2}\;+{\;2y}^{2}\right) \end{array}\right.$$

where $p_{1}$ and $p_{2}$ are the tangential distortion coefficients. Let the coordinates of point p under the ideal, distortion-free imaging model be (*x*,*y*), and the coordinates under the influence of distortion in the actual imaging process be (*x*′,*y*′). The transformation relationship of the coordinates under the influence of both radial and tangential distortions is given by

(24) $$\left\{\begin{array}{c} x^{\prime }=x\left[1\;+k_{1}\left(x^{2}+y^{2}\right)+k_{2}{\left(x^{2}+y^{2}\right)}^{2}\right]+\left(2p_{1}xy+p_{2}\left(3x^{2}+y^{2}\right)\right)\\ y^{\prime }=y[1\;+k_{1}\left(x^{2}+y^{2}\right)+{\;k}_{2}{\left(x^{2}+y^{2}\right)}^{2}]+\left(2p_{2}xy+p_{1}\left(r^{2}+{2y}^{2}\right)\right) \end{array}\right.$$

Lens distortion is modeled using standard radial and tangential components. Radial distortion is represented by coefficients *k*_1_ and *k*_2_ and tangential distortion by *p*_1_ and *p*_2_, which are estimated during camera calibration and used to undistort pixel coordinates before mapping.

### 3.4. Camera Mapping Model Construction and Calibration

The key to constructing the camera mapping model lies in solving for the camera’s extrinsic matrix, distortion coefficients, and intrinsic matrix. These parameters can be computed through camera calibration experiments, thereby constructing a complete camera mapping model that enables the transformation between the aircraft fuselage surface metal corrosion pixel coordinate system and the aircraft fuselage surface world coordinate system.

#### 3.4.1. Monocular Camera Calibration

Monocular camera calibration is the process of estimating the intrinsic and extrinsic parameters of a monocular camera, as well as the distortion coefficients, using a set of known 3D coordinates and their corresponding 2D image coordinates. The main steps are as follows:

Step 1. Intrinsic Calibration:

The camera intrinsic matrix is calculated, which includes parameters such as the focal length, the coordinates of the image center, and the parameters describing radial and tangential distortion. The intrinsic matrix can be determined by capturing images of a checkerboard calibration board, using the 2D coordinates of the chessboard corner points and their corresponding 3D coordinates.

Step 2. Extrinsic Calibration:

The extrinsic matrix describes the relationship between the camera coordinate system and the aircraft fuselage surface world coordinate system. Based on the mapping relationship between the 3D spatial coordinates of the calibration target and their corresponding 2D coordinates in the image, the camera’s rotation matrix and translation vector can be computed.

Step 3. Distortion Coefficient Calibration:

Radial distortion from the lens causes the geometric shape of objects in the image to be stretched or compressed, while tangential distortion results in overall image skew or displacement. By optimizing the distortion coefficients, image correction can be performed to reduce the effects of distortion.

#### 3.4.2. Camera Parameter Estimation

This study extends the camera’s linear imaging model based on the Zhang Zhengyou checkerboard calibration method [39] by incorporating a lens-distortion model. A nonlinear optimization algorithm is employed to improve the accuracy of parameter estimation. The calibration process is divided into two steps:

Step 1. Obtain the camera parameters in the camera mapping linear model without considering lens distortion.

Step 2. Use the undistorted camera parameters as initial values and, based on this, solve for the nonlinear imaging model to determine more accurate camera parameters.

During calibration, the checkerboard plane is treated as Z = 0 in the world coordinate system. A planar homography relates checkerboard world coordinates to image pixel coordinates and serves as the basis for estimating intrinsic and extrinsic parameters.

(25) $$s\left[\begin{array}{c} u\\ v\\ 1 \end{array}\right]=H\left[\begin{array}{c} X_{W}\\ Y_{W}\\ 1 \end{array}\right],\;H\;=\;A[r_{1}\;r_{2}\;t]$$

The camera intrinsic matrix is denoted by ***A***. Because the calibration board is defined on the plane ZW = 0, the planar homography can be written as ***H*** = ***A***[***r***_1_,***r***_2_,***t***] = [***h***_1_,***h***_2_,***h***_3_], where ***r***_1_ and ***r***_2_ are the first two columns of the rotation matrix ***R*** and ***t*** is the translation vector. Each calibration-board pose produces one homography matrix ***H***. Therefore, the mapping between a calibration-plane point and its image point is expressed as

(26) $$H\;=A\left[r_{1},r_{2},t\right]$$

The rotation matrix ***R*** is an orthogonal matrix. Based on the orthogonality of the matrix and the normalization of the unit vectors, the following can be derived:

(27) $$\left\{\begin{array}{c} r_{1}^{T}r_{2}\;=\;0\\ r_{1}^{T}r_{1}\;=\;r_{2}^{T}r_{2} \end{array}\right.$$

Each estimated homography matrix ***H*** provides two independent constraints on the symmetric matrix B, which are used to solve for the intrinsic parameters of the camera.

(28) $$\left\{\begin{array}{c} h_{1}^{T}A^{-T}A^{-1}h_{2}=0\\ h_{1}^{T}A^{-T}A^{-1}h_{1}=h_{2}^{T}A^{-T}A^{-1}h_{2} \end{array}\right.$$

To simplify the calculation, a symmetric matrix **B** is defined as

(29) $$B=A^{-T}A^{-1}=\left[\begin{array}{ccc} b_{11} & b_{12} & b_{13}\\ b_{12} & b_{22} & b_{23}\\ b_{13} & b_{23} & b_{33} \end{array}\right]$$

In matrix **B**, there are six independent elements, which are defined as a six-dimensional vector $b\;=\;{\left[\begin{array}{cccccc} b_{11} & b_{12} & b_{22} & b_{13} & b_{23} & b_{33} \end{array}\right]}^{T}$. Let the *i*-th column vector of matrix H be h_i_, then the following relationship holds:

(30) $$h_{i}^{T}Bh_{j}\;=\;v_{\mathrm{ij}}^{T}b$$

$v_{\mathrm{ij}}^{T}\;=\;\left[h_{i1}h_{j1},h_{i1}h_{j2}\;+\;h_{i2}h_{j1},h_{i2}h_{j2},h_{i1}h_{j3}\;+\;h_{i3}h_{j1},h_{i2}h_{j3}\;+\;h_{i3}h_{j2},h_{i3}h_{j3}\right]$. The constraint equation on the camera’s intrinsic matrix A can be expressed as an equation in terms of the vector ***b***:

(31) $$\left[\begin{array}{c} v_{12}^{T}\\ \left(v_{11}-v_{22}\right) \end{array}\right]b\;=\;0$$

For n calibration images, 2n independent linear constraints can be assembled into a single linear system to estimate the intrinsic parameters. The solution is obtained via least squares with appropriate normalization.

(32) Vb=0

Here, ***V*** is a matrix of size 2n × 6, and when n ≥ 3, a unique solution for the vector b can be determined. This means that when the number of valid calibration board images is greater than or equal to three, the camera parameters can be calibrated.

Assuming n checkerboard calibration images are captured, each with a different pose of the calibration board and containing m corner points, a function is established to minimize the error between the actual pixel coordinates and the ideal coordinates of the corner points. To mitigate the effects of lens distortion, the Levenberg–Marquardt algorithm [40] is used to iteratively optimize the camera’s intrinsic and extrinsic parameters, as well as the distortion coefficients. The objective function f is defined as follows:

(33) $$f\;=\;\min\sum_{i=1}^{n}\sum_{j=1}^{m}{\left\|p_{\mathrm{ij}}\;-p^{\prime }\left(A,k_{1},k_{2},p_{1},p_{2},R_{i},t_{i},P_{j}\right)\right\|}^{2}$$

For the i-th checkerboard image, ***p***_ij_ denotes the observed pixel coordinates of the *j*-th corner point, and ***p***′(·) denotes the corresponding reprojected pixel coordinates obtained from the current camera parameters. Here, ***k***_1_ and ***k***_2_ are the radial distortion coefficients, **p**_1_ and **p**_2_ are the tangential distortion coefficients, **R**_i_ and **t**_i_ denote the rotation matrix and translation vector of the *i*-th calibration image, respectively, and **P**_j_ represents the 3D coordinates of the *j*-th corner point in the world coordinate system.

### 3.5. Published-Method-Inspired Baselines and Fair Comparison Protocol

To quantitatively evaluate the contribution of the proposed physical-surface localization framework, two representative conventional vision-based measurement methods were implemented as comparison baselines: local pixel-to-millimeter scaling and planar homography mapping.

In the local scaling method, detector-generated pixel coordinates were converted into physical coordinates using horizontal and vertical pixel-to-millimeter scale factors obtained from known calibration dimensions. This method does not explicitly account for perspective projection, camera pose, or lens distortion.

Detector-generated bounding-box corners were transformed into physical coordinates using the inverse homography matrix. A conventional calibrated camera-mapping method was additionally implemented as the closest published-method-inspired baseline. Following the general monocular camera-mapping procedure reported in 35, detector-generated bounding-box corners were restored to the original-image coordinate system, corrected using the calibrated lens-distortion parameters, and converted into physical-surface coordinates through the intrinsic and extrinsic camera parameters.

The proposed method used the same calibrated camera-mapping formulation as the conventional mapping baseline. In each fold, the detector-generated corners were first restored to the original-image coordinate system, corrected using the calibrated lens-distortion parameters, and mapped onto the physical surface. The fold-specific physical-plane center shift, estimated exclusively from the other five images, was then subtracted from both mapped corners of the held-out image.

All automatic methods used the same retrained PWDE-YOLOv8n outputs, locked held-out images, calibration observations, manually measured physical-coordinate references, and evaluation metrics. All methods used the same input images and detector outputs. Runtime was measured with a batch size of one after 50 warm-up iterations, followed by 200 repeated timing iterations. GPU synchronization was performed immediately before and after timing. For physical-localization methods, both mapping-only runtime and complete end-to-end runtime were reported. Mapping-only runtime included coordinate restoration, detector-center correction when applicable, distortion correction, and physical-coordinate conversion, whereas end-to-end runtime additionally included image acquisition, image enhancement, detector inference, and non-maximum suppression.

### 3.6. First-Order Analytical Validity Range of the Local-Planar Approximation

The proposed camera-mapping model does not approximate the entire aircraft fuselage as a single plane. Instead, it represents a limited inspection region using the tangent plane at the center of the local fuselage surface. To analytically estimate the additional localization error introduced by this approximation, the local fuselage cross-section was represented as a circular arc with radius ρ, whose axis was assumed to be parallel to the local Y-direction.

For a local inspection region with physical width *W*, the normal deviation between the curved surface and the tangent plane at position x is

(34) $$s(x)=\rho -\sqrt{\rho^{2}-x^{2}},\;-\frac{W}{2}\leq x\leq \frac{W}{2}$$

The maximum sagitta occurs at the edge of the inspected region:

(35) $$s_{max}\;=\;\rho -\sqrt{\rho^{2}-{\left(\frac{W}{2}\right)}^{2}}$$

When the viewing direction forms an angle *θ* with the local surface normal, the first-order curvature-induced axial error is estimated as

(36) $$e(x)=s(x)tan\theta ,{\;e}_{max}=s_{max}tan\theta$$

The mean axial error over the inspected width is estimated as

(37) $$\overset{-}{e}=\frac{\tan\theta }{W}\int_{-W/2}^{W/2}\left[\rho -\sqrt{\rho^{2}-x^{2}}\right]dx$$

For a W-wide rectangular localization region subjected to an axial displacement *e*_max_, the corresponding first-order IoU loss is estimated as

(38) $$\Delta IoU=100[1-\frac{W-e_{max}}{W+e_{max}}]$$

This analysis provides a first-order analytical error bound rather than a numerical ray–cylinder intersection using the actual camera intrinsic and extrinsic parameters. The sensitivity analysis considered curvature radii of 1500–4000 mm, localization-region widths of 100–200 mm, and viewing angles of 15–30°. The working distance of 500–600 mm describes the representative acquisition configuration but does not explicitly enter the adopted first-order error bound. The allowable curvature-induced additional axial error was set to

(39) $$e_{allow}=1.0\;mm$$

## 4. Experiments and Results

**Figure 5:**
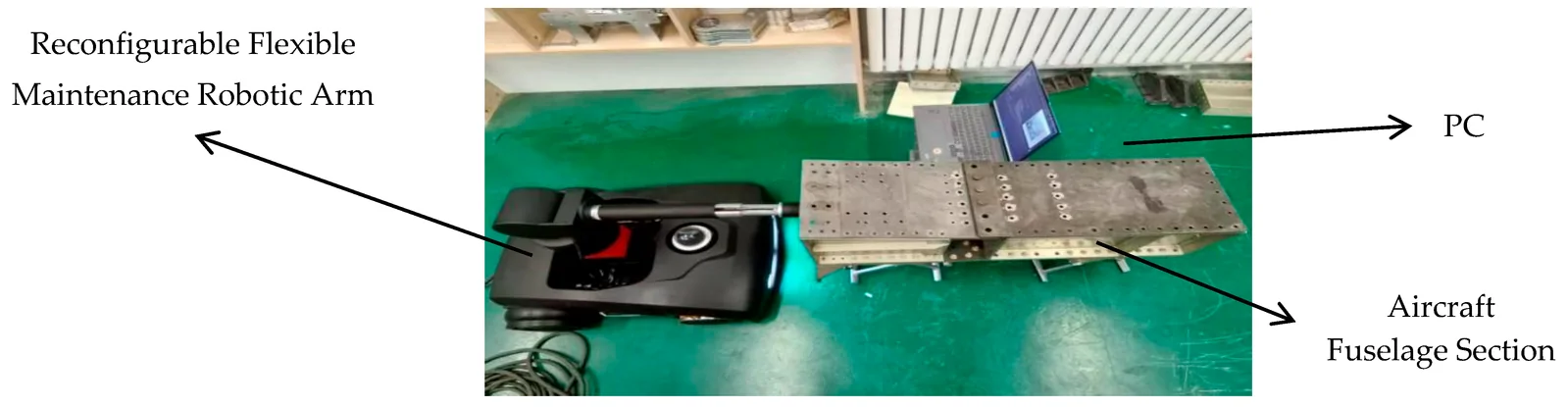
Integrated corrosion localization platform.

To achieve high-precision and near-real-time physical localization of metal corrosion on the aircraft fuselage surface, this study developed a camera-calibrated vision measurement platform integrating PWDE-YOLOv8n-based corrosion perception, Cross-Validated Detector-Center Correction, and a calibrated camera mapping model. As shown in Figure 5, the platform integrates key components, including a reconfigurable flexible maintenance robotic arm, camera modules, lenses, and illumination devices. The corrosion perception module identifies corrosion regions in the image domain, while the camera mapping model converts the detected corrosion boxes into physical-surface coordinates. The Cross-Validated Detector-Center Correction module estimates the fold-specific physical-plane center shift from the other five images and applies it unchanged to both mapped corners of the held-out image after original-coordinate restoration, lens-distortion correction, and ray-to-surface mapping. Image-formation edge spreading is discussed as an upstream source affecting boundary observability but is not treated as being equivalent to the final detector-output box bias.

(1) Industrial Camera Module: The core device for image acquisition. This study employs a USB 3.0 camera known for its high image quality and strong anti-interference capabilities, making it suitable for complex environments. Considering the characteristics of both the internal and external surfaces of the aircraft fuselage and the low-light conditions of maintenance holes, the HBVCAM-4K2353V22 camera module was selected to meet the high-resolution imaging requirements.

(2) Lens: The lens determines the imaging quality and must have low distortion, high resolution, and adaptability to low-light conditions in maintenance holes. Based on the CCD camera parameters and imaging requirements, the BT-23C0814MP5 large-aperture lens from Vision Intelligence was chosen to enhance image brightness under weak lighting conditions.

(3) Light Source: To ensure clear details of the defect areas, a ring-shaped LED light source is embedded around the CCD camera lens, improving illumination uniformity. Additionally, to meet the weight-bearing requirements of the robotic arm’s end effector, the MV-RL51X25W-F ring light source from Vision Intelligence was selected.

The key hardware parameters related to image acquisition and imaging quality in the experimental system are shown in Table 4.

All experiments were conducted on a Windows 10 workstation equipped with an Intel Xeon Platinum 8370C CPU, 256 GB RAM, and an NVIDIA GeForce RTX 4090 GPU. The detector was implemented in PyTorch 1.13.1 with CUDA 11.6. Unless otherwise specified, runtime was measured per image, and the reported end-to-end time includes image acquisition, enhancement, detection, and physical localization.

### 4.1. Camera Calibration Experiment and Physical-Coordinate Reference

For each corrosion box, the predicted upper-left and lower-right corners are mapped from image coordinates to the fuselage surface coordinate system. We report two localization metrics. First, the axial localization errors are defined as the absolute errors of the mapped corner coordinates along the surface X- and Y-axes. Second, IoU is computed between the projected predicted box and the projected ground-truth box on the same local surface plane. In the current implementation, the projected regions are represented as axis-aligned rectangles in the surface coordinate system for IoU computation.

The calibration board used in this experiment has dimensions of 100 mm × 100 mm, with a checkerboard pattern consisting of 12 × 11 inner corner points. The size of each checkerboard square is 6 mm × 6 mm. A total of 20 checkerboard images were captured at different poses, yielding up to 2640 corner observations when all images were valid.

**Figure 6:**
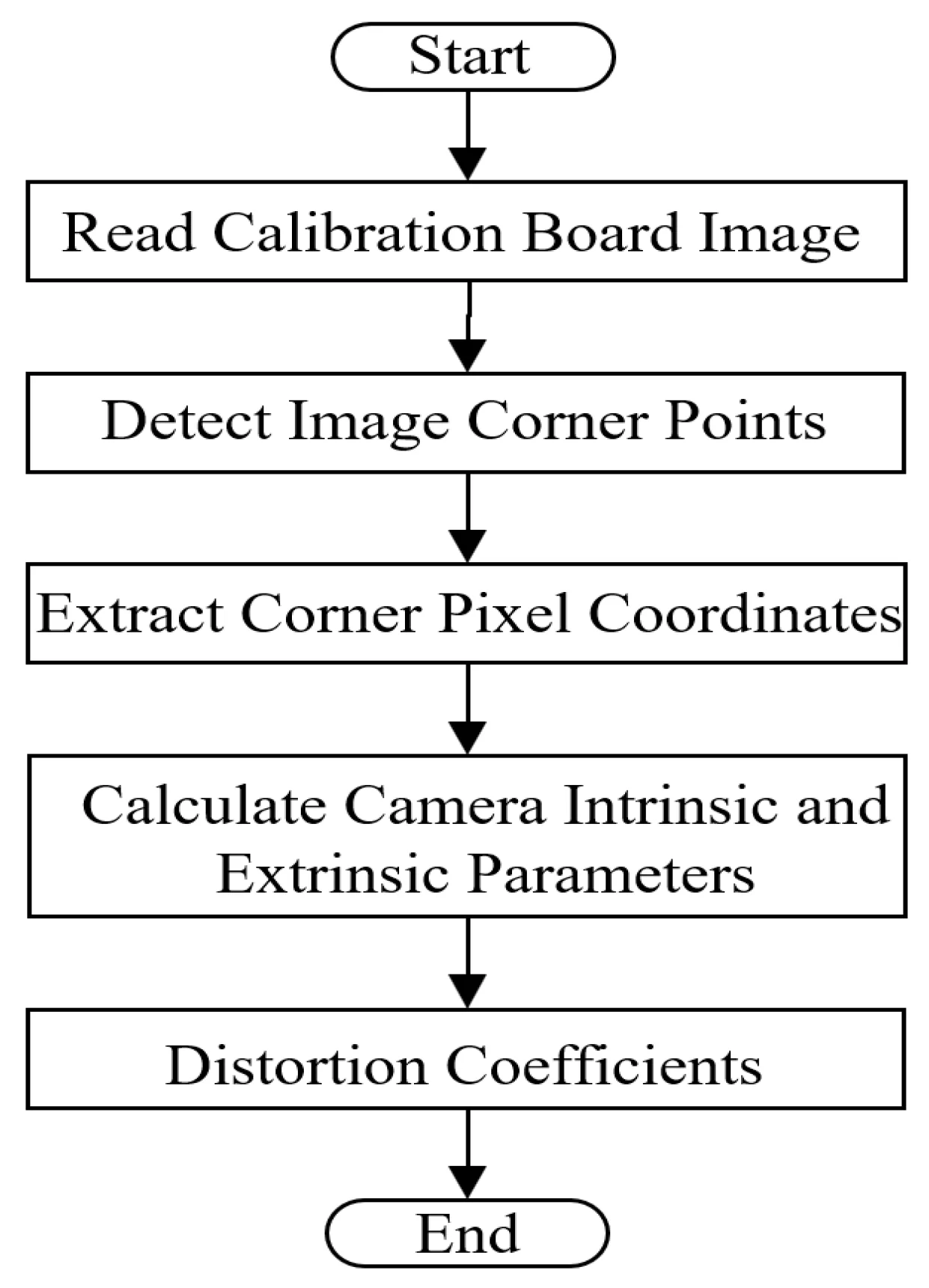
Camera calibration workflow.

Checkerboard corner locations were detected automatically and then used directly as inputs to the subsequent nonlinear calibration optimization, without additional subpixel refinement. Reprojection error is defined as the Euclidean distance between the observed checkerboard corner and its reprojected location in the image plane. In this study, the reprojection error is reported in pixel units. The per-image average reprojection errors for the 20 calibration images are distributed between 0.033 and 0.035 pixels, with an overall mean value of 0.033 pixels. The distortion parameters follow the OpenCV pinhole-camera model in normalized image coordinates; therefore, their numerical scales should not be interpreted directly as pixel-domain displacements. The calibration process is shown in Figure 6. Accordingly, the reported tangential distortion coefficients should be interpreted within the adopted normalized-coordinate convention rather than as direct pixel-domain offsets.

**Figure 7:**
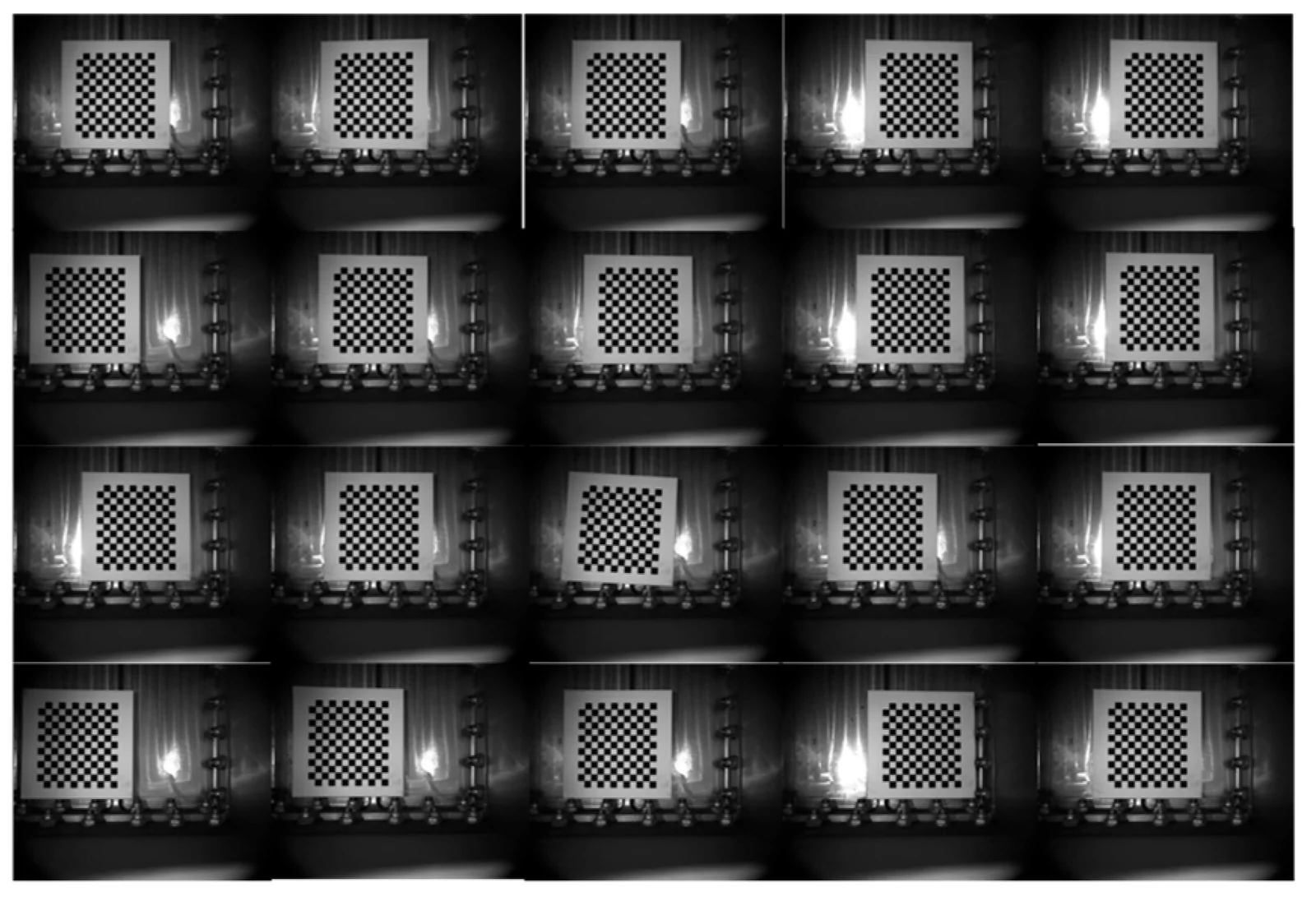
Calibration-board images captured at different poses.

As shown in Figure 7, a total of 20 images of the checkerboard calibration board were captured, each at a different pose.

**Figure 8:**
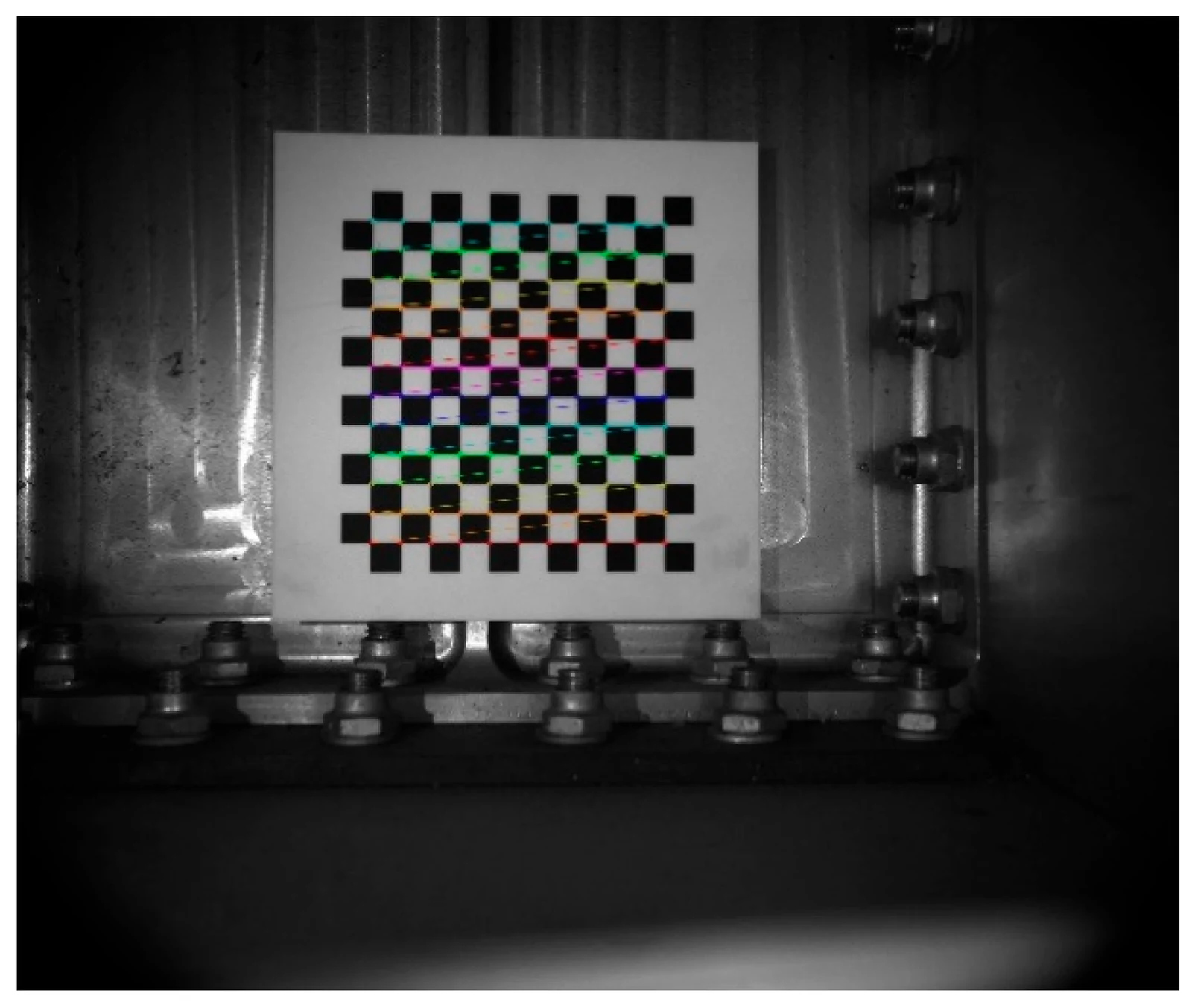
Detected corner points in a calibration image.

The aircraft fuselage surface metal corrosion pixel coordinate system has its origin at the top-left corner of the image. The x-axis values increase horizontally from left to right, while the y-axis values increase vertically from top to bottom. The top-left inner corner of the checkerboard calibration board is set as the origin of the aircraft fuselage surface world coordinate system for calibration. Figure 8 illustrates the detection of the inner corner points of the checkerboard calibration board in the captured images during the calibration process.

The calibrated intrinsic parameters are:

Focal lengths: $f_{c}\;=\;\left[f_{x},f_{y}\right]\;=\;\left[2426.2685,2426.291\right]$;

Principal point coordinates: $\left[c_{x},c_{y}\right]\;=\;\left[1252.27807,1018.87380\right]$;

Radial distortion coefficients: $\left[k_{1},k_{2}\right]\;=\;\left[-0.0682786619,0.210918559\right]$.

The distortion parameters follow the OpenCV pinhole-camera model in normalized image coordinates; therefore, their numerical scales should not be interpreted directly as pixel-domain displacements.

Tangential distortion coefficients: $p_{1}\;=\;-0.0000538702879,\;p_{2}=0.0324208284$.

The intrinsic matrix is given by

$$K=\left[\begin{array}{ccc} f_{x} & \gamma & c_{x}\\ 0 & f_{y} & c_{y}\\ 0 & 0 & 1 \end{array}\right]=\left[\begin{array}{ccc} 2426.26850 & 0 & 1252.27807\\ 0 & 2426.29100 & 1018.87380\\ 0 & 0 & 1 \end{array}\right]$$

The rotation matrices and translation vectors computed for the first three calibration-board images are as follows:

$$R_{1}\;=\;\left[\begin{array}{ccc} 0.000356 & -0.998064 & -0.062188\\ 0.999742 & -0.001056 & 0.022669\\ -0.022691 & -0.062180 & 0.997807 \end{array}\right],{\;T}_{1}\;=\;\left[\begin{array}{c} 11.34643455\\ -56.9084829\\ 238.17702624 \end{array}\right]$$

$$R_{2}=\left[\begin{array}{ccc} -0.001601 & -0.999776 & -0.021117\\ \;0.998360\; & -0.002807 & 0.057183\\ -0.057229 & -0.020991 & 0.998140 \end{array}\right],{\;T}_{2}=\left[\begin{array}{c} -31.31545069\\ -56.74451853\\ 235.15777686 \end{array}\right]$$

$$R_{3}=\left[\begin{array}{ccc} -0.051524 & -0.998383 & -0.024021\\ \;0.995904 & -0.053156 & 0.073138\\ -0.074296 & -0.020154 & 0.997033 \end{array}\right],\;T_{3\;}=\left[\begin{array}{c} -1.69910812\\ -53.11163776\\ 235.47533685 \end{array}\right]$$

For the *i*-th feature point, the difference between the actual pixel coordinates *u*_i_, *v*_i_ and the pixel coordinates *u*_i_, *v*_i_ computed by the camera mapping model is referred to as the reprojection error. The accuracy of camera calibration can be evaluated by calculating the average of the reprojection errors for all corner points. The calculation formula is as follows:

(40) $$e\;=\;\frac{1}{N}\sum_{i=1}^{N}\sqrt{{\left(u_{i}\;-\;{u_{i}}^{\prime }\right)}^{2}\;+\;{\left(v_{i}\;-\;{v_{i}}^{\prime }\right)}^{2}}$$

where *N* is the total number of checkerboard feature points used in calibration, (*u*_i_,*v*_i_) denotes the observed pixel coordinates of the *i*-th corner point, and (*u*_i_′,*v*_i_′) denotes the corresponding reprojected pixel coordinates obtained from the calibrated camera model.

**Figure 9:**
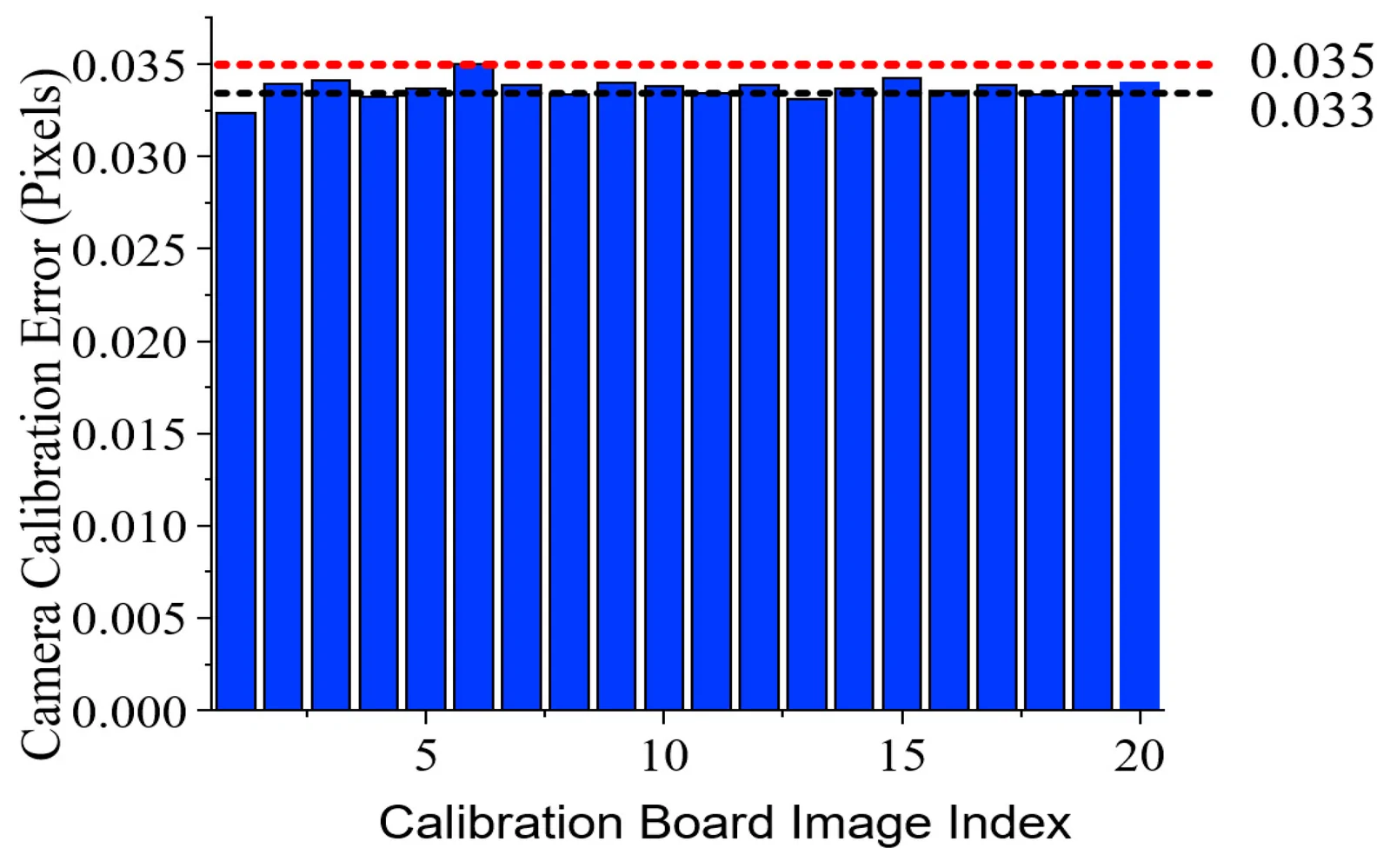
Per-image average reprojection errors for the 20 calibration images.

As shown in Figure 9, the per-image average reprojection errors for the 20 calibration images are tightly distributed between 0.033 and 0.035 pixels. The overall mean reprojection error is 0.033 pixels, and the maximum reported reprojection error is 0.035 pixels. Here, the reprojection error is reported in pixel units and is defined as the Euclidean distance between the observed checkerboard corner and its reprojected location after the final distortion-aware nonlinear parameter optimization.

**Figure 10:**
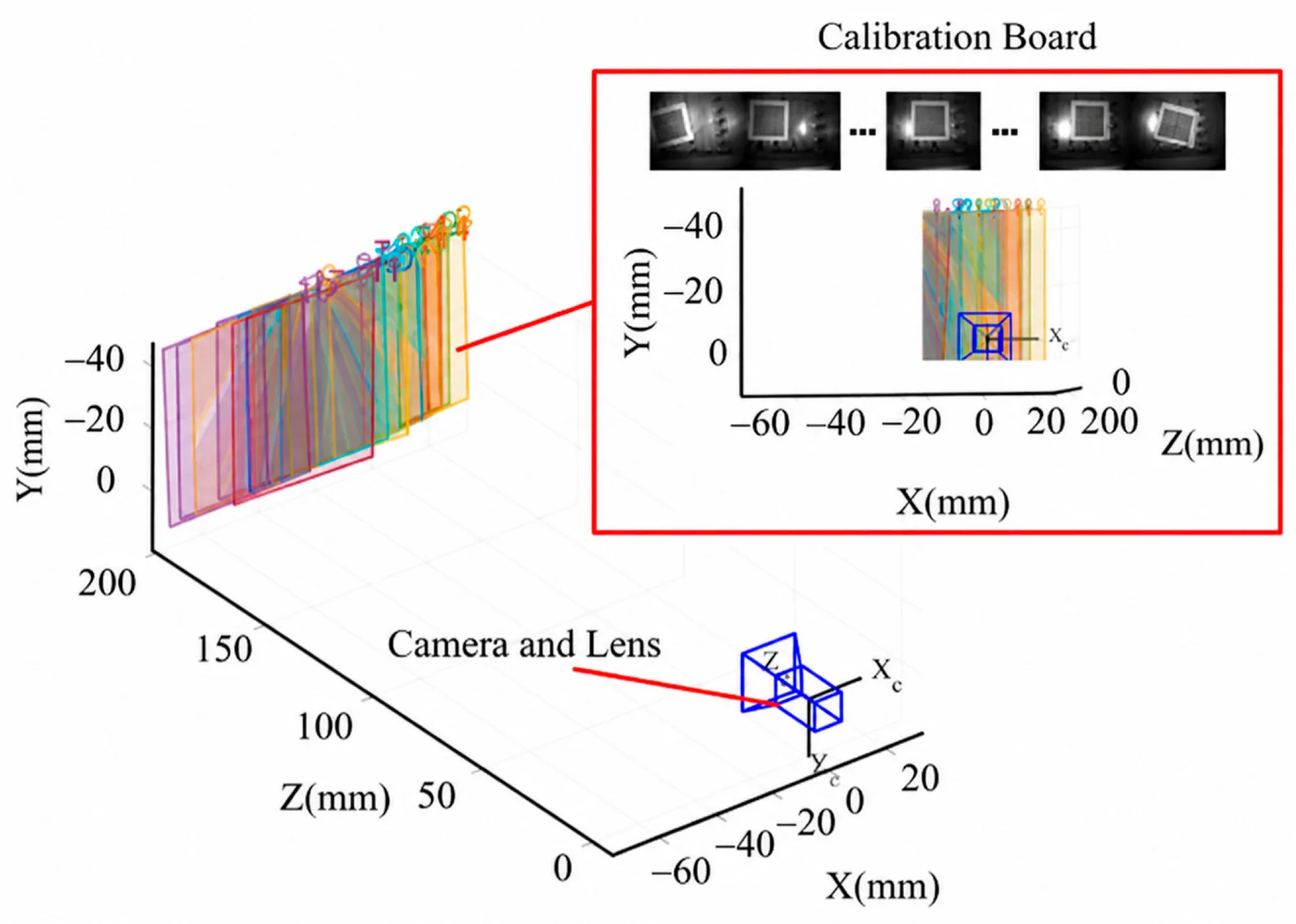
Spatial poses of the calibration board relative to the camera.

Figure 10 illustrates the different poses of the calibration-board images and the relative position between the camera and the board.

Step 1. System setup and positioning.

Communication between the host computer and the robotic arm was established to control the servos, enabling precise positioning of the robotic arm near the aircraft fuselage surface. The industrial camera-lens module mounted on the end effector was then adjusted to an appropriate pose, ensuring that the region of interest was centered within the camera’s field of view.

Step 2. Image acquisition and corrosion detection.

A complete and clear image of the aircraft fuselage surface was captured. The PWDE-YOLOv8n model was used to identify metal corrosion on the surface. The output included the location information of the corrosion in the image, which was then converted to the pixel coordinates of the corrosion bounding-box corners.

Step 3. Camera calibration and mapping model establishment.

With the camera position fixed, the checkerboard calibration board was placed on the local inspection region. The tangent plane defined by the calibration board was treated as Z = 0. This approximation was applied only when the local curvature radius and inspection-region width satisfied the applicability criterion defined in Section 3.6.

Step 4. World-coordinate origin marking and manual measurement.

A standard 0-grade gauge block (with a precision of 0.001 mm, length of 20 mm, width of 35 mm, and height of 9 mm) was placed at the top-left inner corner of the checkerboard calibration board. A removable marker pen was used to mark the origin of the aircraft fuselage surface world coordinate system. The calibration board was then removed, ensuring that the manually measured origin aligned with the world coordinate system origin. Subsequently, a removable marker pen and engineering resolution of 0.1 mm were used to draw a rectangular box to define the corrosion location. The coordinates of the upper-left and lower-right corners of each corrosion area were recorded in the X and Y directions of the aircraft fuselage surface world coordinate system, with the gauge block position serving as the origin.

Step 5. Physical-coordinate computation.

The pixel coordinates of the upper-left and lower-right corners of the corrosion bounding box were input into the camera mapping model, and the corrosion location on the physical surface of the aircraft fuselage was computed.

Step 6. Error computation.

The computed coordinates were compared with the manually measured coordinates to calculate the axial localization errors and the Intersection over Union (IoU).

The physical-coordinate reference was obtained by manual marking and engineering measurement. A 0-grade gauge block used for coordinate-origin transfer was used to transfer the world-coordinate origin from the checkerboard reference point. After the calibration board was removed, the upper-left and lower-right corners of each corrosion region were manually marked, and their X- and Y-axis coordinates were measured using an engineering tape with a resolution of 0.1 mm. Therefore, the reported localization error includes both algorithmic error and manual measurement uncertainty.

The uncertainty sources associated with the manually established physical-coordinate references are summarized in Table 5.

The uncertainty budget shows that the 0.1 mm tape resolution represents only one component of the reference uncertainty. Repeated corrosion-boundary marking, coordinate-origin transfer, axis establishment, and operator-dependent boundary interpretation were also included in the combined uncertainty.

For each mapped corrosion bounding box, the absolute axial localization errors were calculated for the upper-left and lower-right corner coordinates along the local surface X- and Y-directions. In addition to the mean absolute axial error, the localization performance was summarized using the standard deviation, median, interquartile range, root-mean-square error (RMSE), and maximum axial error.

$$RMSE=\sqrt{\frac{1}{N}\sum_{i=1}^{N}{\left(\hat{q_{i}}-q_{i}^{ref}\right)}^{2}}$$

where ${\hat{q}}_{i}$ denotes the mapped physical coordinate, $q_{i}^{\mathrm{ref}}$ denotes the corresponding manually measured reference coordinate, and N denotes the total number of evaluated axial coordinates. For each corrosion bounding box, four axial coordinates were evaluated, including the X- and Y-coordinates of the upper-left corner and those of the lower-right corner. Because one image could contain multiple corrosion boxes, observations originating from the same image were not treated as completely independent samples. The 95% confidence intervals of the mean absolute axial error and projected bounding-box IoU were therefore estimated using 1000 image-level cluster-bootstrap resamples. During each resampling iteration, images were sampled with replacement, and all corrosion boxes belonging to a selected image were retained together. The proportions of axial localization errors not exceeding 1, 2, 3, and 5 mm were also calculated to provide an intuitive description of maintenance-oriented localization performance. The cluster-bootstrap confidence intervals describe sampling variability across held-out images, whereas the Monte Carlo intervals describe uncertainty introduced by the manually established physical references. These two intervals were therefore reported separately.

### 4.2. Cross-Validated Detailed Physical-Localization Evaluation

Detailed physical localization was evaluated on six calibrated images acquired in six sessions, containing 21 corrosion boxes and 84 evaluated axial coordinates. Because of the limited size of this metrology cohort, a six-fold leave-one-image-out procedure was used. In each fold, the center-shift parameters were estimated from the other five images and applied unchanged to the held-out image. The evaluated image therefore did not contribute to its own correction parameters. The results should be interpreted as a preliminary cross-validated metrology assessment rather than statistically comprehensive external validation.

The center-corrected mapping achieved a mean absolute axial error of 0.641 mm, with a 95% image-cluster bootstrap confidence interval of 0.571–0.708 mm. The RMSE, median error, and maximum error were 0.809, 0.600, and 3.262 mm, respectively. The projected physical-plane bounding-box IoU was 87.12%, and 98.81% of the evaluated axial errors were within 3 mm.

The combined standard uncertainty of the manually established physical-reference coordinates was 0.187 mm, corresponding to an expanded uncertainty of 0.374 mm at k = 2. After 10,000 Monte Carlo trials, the propagated mean absolute error was 0.656 mm, with a 95% interval of 0.618–0.693 mm. The corresponding projected IoU was 86.83%, with a 95% interval of 86.11–87.54%. The reference uncertainty was smaller than, but not negligible relative to, the observed localization-error scale.

### 4.3. Practical Utility of Cross-Validated Detector-Center Correction

The theoretical analysis in Section 2.5.1 distinguishes image-formation edge spreading from detector-output box bias. The former describes the finite-width intensity transition generated during optical imaging and sensor sampling, whereas the latter is measured from the coordinate residual between predicted and manually annotated bounding boxes after detector inference.

The proposed correction module operates on physical-plane detector-center bias. In each fold, the center-shift parameters were estimated from the other five images and fixed before application to the held-out image. The ablation study compared raw detector boxes, center-shift correction only, size correction only, and combined center-and-size correction using out-of-fold results. Center-shift correction only achieved the best overall performance and was retained as the final strategy. Table 6 summarizes the main sources of detector-output box uncertainty and the corresponding handling strategy adopted in framework.

The detector-center correction may further be conditioned on reflection coverage, mean intensity, local contrast, and detector confidence rather than using a single condition-independent center-shift model. For every matched box in the five parameter-estimation images, the detector-generated corners were first restored to the original-image coordinates, corrected for lens distortion, and mapped onto the physical surface. The physical-plane center residuals were then calculated between the mapped detector-box center and the corresponding manually established physical-reference center. The median horizontal and vertical residuals were used as the fold-specific physical-plane center-shift parameters. The resulting shift was applied unchanged to both mapped corners of the held-out image. Table 7 lists the physical-plane center-shift parameters obtained from the six-fold leave-one-image-out cross-validation.

The correction parameters in each row were estimated exclusively from the other five images and were applied unchanged to the corresponding held-out image. The equivalent physical shifts were subtracted from both mapped corners; therefore, the physical width and height of each predicted box were preserved. No annotation, physical reference, or localization error from the held-out image was used for parameter estimation. Table 8 presents the ablation study of detector-center and size correction under six-fold leave-one-image-out evaluation.

Compared with the raw detector boxes, the six-fold leave-one-image-out center-shift correction reduced the mean absolute axial error from 2.179 mm to 0.641 mm and increased the projected physical-plane bounding-box IoU from 63.45% to 87.12%. For each held-out image, the correction parameters were estimated exclusively from the other five images and were then applied unchanged to the held-out image. They were not refitted or adjusted using any annotation, physical-coordinate error, or evaluation result from that image. The image-level paired reduction in mean absolute axial error was 1.538 mm, with a 95% image-cluster bootstrap confidence interval of 1.203–1.785 mm. The corresponding IoU improvement was 23.67 percentage points, with a 95% confidence interval of 15.63–27.67 percentage points. Size correction alone did not improve the mean absolute axial error, whereas combining center and size corrections resulted in an MAE of 0.664 mm and an IoU of 86.66%. Therefore, center-shift correction only was retained as the final correction strategy.

### 4.4. Detector-Based Corrosion Localization in Image Coordinates

**Figure 11:**
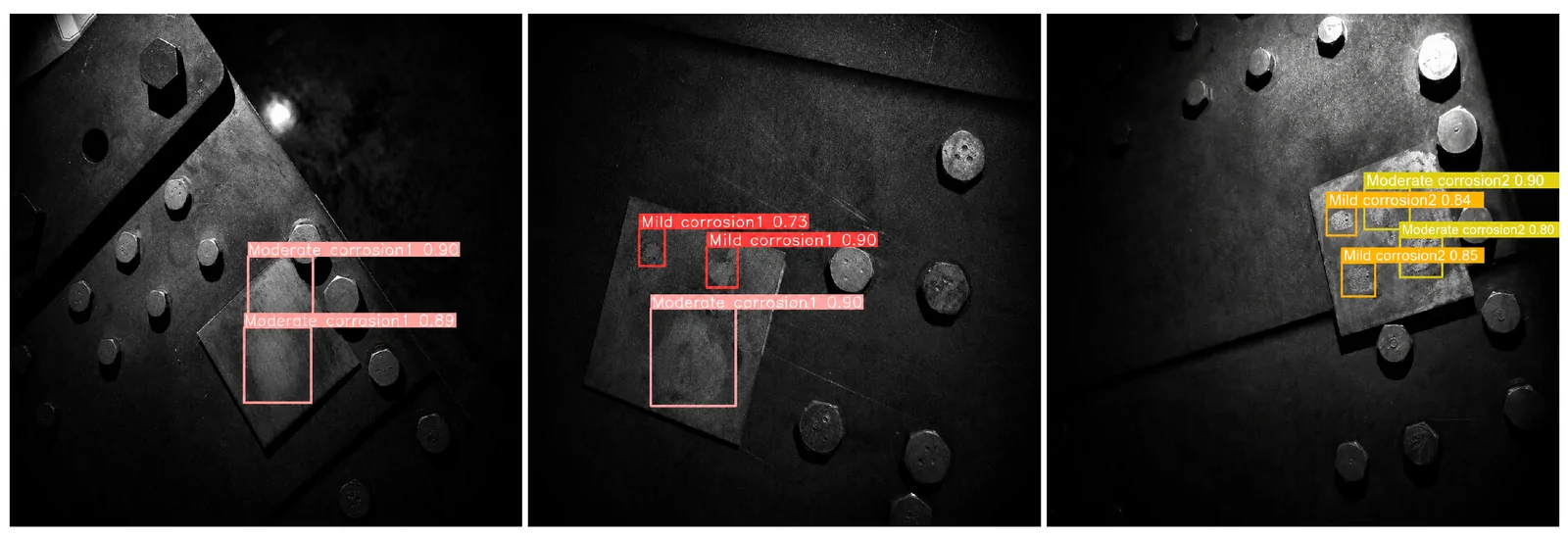
Example pixel-level detection results from PWDE-YOLOv8n [27].

The PWDE-YOLOv8n-based corrosion perception module outputs the bounding-box coordinates of each corrosion region in detector-input space, including the upper-left corner (x_1_, y_1_) and the lower-right corner (x_2_, y_2_). Non-maximum suppression is then applied to remove redundant predictions and retain the final detector-generated box for each corrosion region. Because the retained box may be affected by detector-output boundary uncertainty, its coordinates are not directly interpreted as physical corrosion boundaries. Instead, the coordinates are first restored to the original-image coordinate system according to the preprocessing transform. As shown in Figure 11, these restored corner coordinates are used as the input to the calibrated camera mapping model for subsequent physical localization on the local fuselage surface plane.

### 4.5. Conversion of Detector-Generated Corrosion Location Information

The corrosion perception results output by PWDE-YOLOv8n include the corrosion class, confidence score, and the coordinates of the bounding-box center together with the width w and height h of the bounding box. The pixel coordinates of the detector-generated corrosion box use the top-left corner of the image as the origin, with pixel units. The x-coordinate increases horizontally from left to right, and the y-coordinate increases vertically from top to bottom. Because the detector-generated box may exhibit center displacement or size bias relative to the manually annotated reference. In each fold, the center-shift parameters were fixed after estimation from the other five images and then applied unchanged to the held-out image. No predicted box, annotation, physical-coordinate reference, or localization error from the held-out image was used to estimate or modify its correction parameters.

The corrosion localization method based on the camera mapping model requires obtaining the location information of corrosion in image coordinates. The localization of the recognition box must rely on the corner point coordinates. To do this, the coordinates of the upper-left corner and the lower-right corner of the corrosion recognition box must be obtained and input into the camera mapping model for coordinate transformation to determine the corrosion location on the physical surface of the aircraft fuselage. The conversion of the center coordinates, width w, and height h to the corner coordinates of the corrosion recognition box is given by:

(41) $$u_{1}=\;u_{\mathrm{center}}-\frac{w}{2}$$

(42) $$v_{1}=v_{\mathrm{center}}-\frac{h}{2}$$

(43) $$u_{2}=u_{\mathrm{center}}+\frac{w}{2}$$

(44) $$v_{2}=v_{\mathrm{center}}+\frac{h}{2}$$

To illustrate this conversion procedure, six images from the corrosion localization experiment were selected. For each detected corrosion region, the center coordinates, box dimensions, converted upper-left and lower-right corner coordinates, and the corresponding sample categories are listed in Table 9.

### 4.6. Physical-Surface Localization Results and Analysis

The converted upper-left and lower-right corner coordinates obtained in Table 9 were input into the camera mapping model to estimate the corresponding corrosion locations on the aircraft fuselage surface. The localization results are summarized in Table 10.

For each corrosion instance in Table 10, the physical coordinates of the upper-left and lower-right corners are reported for both the manually measured reference and the coordinates predicted by the camera mapping model. The corresponding axial localization errors along the surface coordinate axes are also listed for the two corners.

Coordinates are reported as (X1,Y1) and (X2,Y2), corresponding to the two opposite corners of each corrosion bounding box. The corrected coordinates are out-of-fold results obtained using the center-shift parameters listed in Table 7. For each held-out image, the correction parameters were estimated exclusively from the other five images. The axial localization errors are absolute coordinate differences. The expanded uncertainty of the manually established reference coordinates was 0.374 mm at k = 2. Across the 84 evaluated axial coordinates, the cross-validated center-corrected results achieved a mean absolute error of 0.641 mm, an RMSE of 0.809 mm, a median error of 0.600 mm, and a maximum error of 3.262 mm. A total of 98.81% of the axial localization errors were within 3 mm.

**Figure 12:**
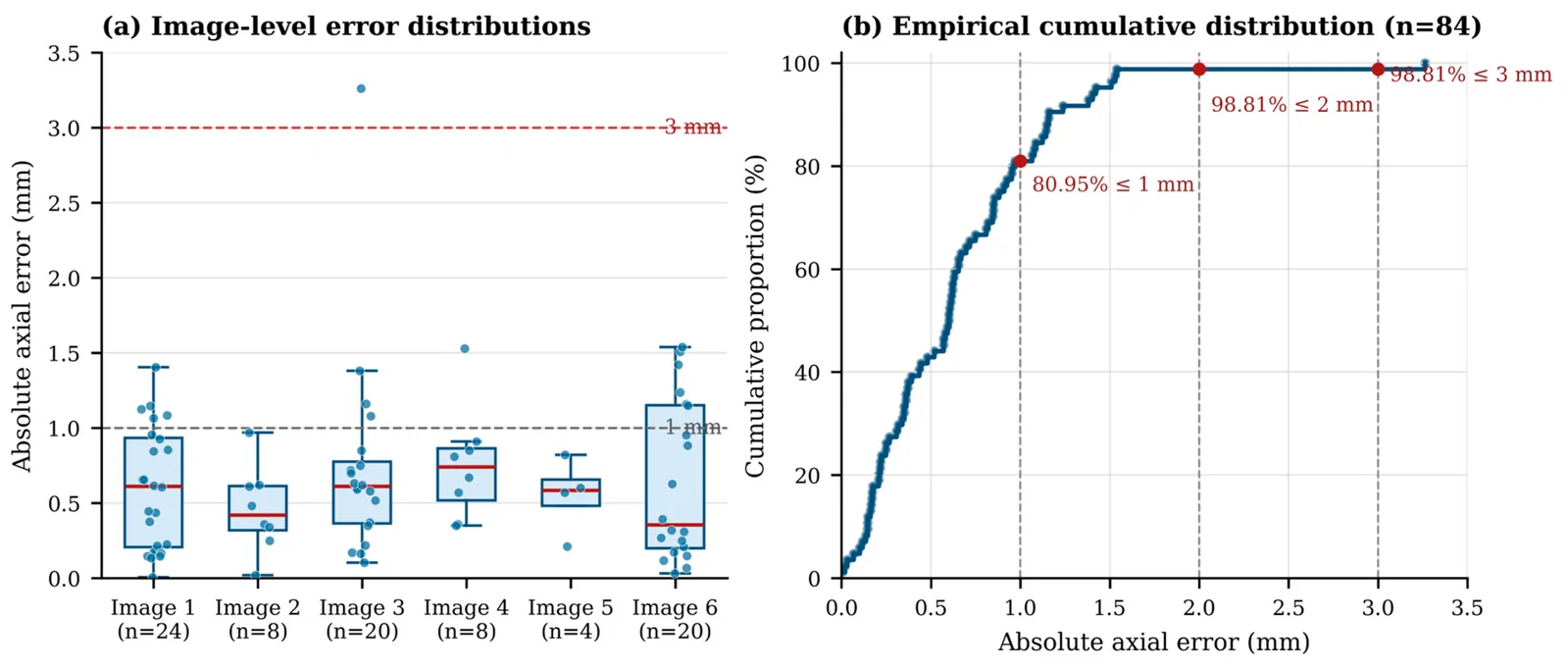
Distribution of cross-validated physical-surface localization errors: (**a**) image-level axial-error distributions with individual observations; (**b**) empirical cumulative distribution of the 84 axial localization errors.

Among all evaluated samples, most axial errors remained within 3 mm. The interpretation of individual sub-millimeter error values was limited by the expanded uncertainty of the manually established reference coordinates; therefore, the overall performance was assessed primarily using the mean, median, RMSE, confidence intervals, and uncertainty-propagated metric ranges. These quantitative results provide the basis for the subsequent visualization of axial localization errors and the IoU-based evaluation of corrosion-region localization accuracy. The distribution of the axial localization errors is shown in Figure 12.

To further illustrate the robustness and variability of the localization results beyond the aggregate mean value, the image-level error distribution and cumulative error proportion are shown in Figure 12.

Figure 12 shows that 68 of the 84 axial localization errors (80.95%) were within 1 mm, while 83 errors (98.81%) were within 2 mm and also within 3 mm. Only one axial coordinate exceeded 3 mm, with a maximum error of 3.262 mm in held-out image 3. The image-level distributions therefore indicate that the corrected localization errors were generally concentrated below 1 mm, although one difficult corrosion box produced a larger residual error.

The present six-image cross-validated metrology cohort primarily contains individually identifiable pitting-corrosion regions. Therefore, the reported results do not yet quantify cases in which pitting corrosion, crevice corrosion, coating damage, scratches, or crack-like features coexist or overlap within the same local region. In such cases, class competition, overlapping visual patterns, and non-maximum suppression may cause multiple defects to be merged into one bounding box or one damage type to suppress another. A single axis-aligned physical bounding box may consequently overestimate the affected surface area or fail to preserve the physical boundaries of the individual damage types.

The current results are also limited to the curvature operating range defined in Section 4.9. For surfaces whose curvature exceeds this range, the difference between the assumed ray–plane intersection and the actual ray–surface intersection increases. Therefore, the errors reported in this section should not be extrapolated to highly curved fuselage regions without replacing the tangent-plane model with a curved-surface representation.

The proposed corrosion localization method for aircraft fuselage surface metal corrosion exhibits certain localization errors. From both theoretical and methodological perspectives, the primary sources of these errors include deviations between the corrosion bounding boxes predicted by the PWDE-YOLOv8n model and the corresponding ground-truth bounding boxes, as well as inaccuracies in the camera parameters and distortion coefficients estimated during the camera calibration process. To further evaluate the accuracy of corrosion region localization, IoU was adopted to verify the localization accuracy of corrosion regions. The IoU values of all corrosion regions in the six held-out metrology images were calculated, and the average IoU values are reported in Table 11.

The overall surface-plane IoU was calculated over all corrosion boxes rather than by averaging the six image-level values. The average processing time per image was calculated over the six held-out metrology images. The runtime results are summarized in Table 12.

Runtime was evaluated on a Windows 10 workstation equipped with an Intel Xeon Platinum 8370C CPU, 256 GB RAM, and an NVIDIA GeForce RTX 4090 GPU. The detector was implemented in PyTorch 1.13.1 with CUDA 11.6. Unless otherwise specified, runtime was measured per image. We report both detector inference time and end-to-end pipeline time. The detector inference time corresponds to the PWDE-YOLOv8n forward pass, whereas the end-to-end runtime additionally includes image acquisition, image enhancement, and physical-coordinate computation. Under this setting, the average end-to-end processing time is 41.05 ms per image, including 16.78 ms for image acquisition, 8.17 ms for image enhancement, 9.58 ms for corrosion detection, and 6.52 ms for physical localization.

The physical-coordinate ground truth was obtained using manual marking and engineering tape measurement. Therefore, the reported localization error contains both algorithmic error and manual measurement uncertainty. In the current study, the latter was partially controlled through the use of a 0-grade gauge block with a precision of 0.001 mm for origin transfer and a measurement tape with a resolution of 0.1 mm for corrosion-box dimension reading.

### 4.7. Quantitative Benchmarking Against Conventional and Published Vision-Localization Methods

#### 4.7.1. Same-Platform Quantitative Comparison

The proposed physical-surface localization framework was quantitatively compared with local pixel-to-millimeter scaling and planar homography mapping using the same locked held-out images and detector-generated bounding boxes. The quantitative results are summarized in Table 13.

As shown in Table 13, the proposed center-corrected camera-mapping framework achieved a mean absolute axial error of 0.641 mm, compared with 30.694 mm for local pixel-to-millimeter scaling, 0.616 mm for planar homography mapping, and 2.179 mm for the conventional calibrated camera-mapping baseline. Relative to the conventional calibrated mapping method, the Cross-Validated Detector-Center Correction reduced the mean absolute axial error by 70.58% and increased the projected bounding-box IoU by 23.67 percentage points, while introducing an additional mapping time of only 0.04 ms per image. These results indicate that the fold-specific detector-center correction provides a measurable accuracy benefit beyond conventional camera calibration and image-to-plane mapping.

#### 4.7.2. Contextual Comparison with Published Studies

In addition to the same-platform reimplemented baselines, the proposed framework was compared contextually with representative published studies on aircraft corrosion inspection, image-domain corrosion perception, monocular visual positioning, and camera-mapping-based defect localization. Because these studies used different datasets, sensing configurations, hardware platforms, and output definitions, their reported values are presented as contextual references rather than as a direct numerical ranking. Table 14 summarizes the contextual comparison between our proposed framework and previously reported representative studies.

Because the published studies used different objects, datasets, output definitions, reference systems, and hardware platforms, the reported values are provided for quantitative context rather than direct performance ranking.

### 4.8. Deployed-System Validation

To examine the engineering deployability of the inspection pipeline, ten additional online images acquired under practical inspection conditions and successfully detected by the deployed system were used as an engineering replication set. These images were not used to estimate, select, or tune any detector-center correction parameters. To avoid transferring the fold-specific parameters beyond their corresponding cross-validation folds, physical localization of the ten online images was performed using the calibrated camera-mapping procedure without detector-center correction. Because the localization set contained only successfully detected cases, it should be interpreted as an engineering replication rather than an unbiased external detection or metrology test. The average surface-plane IoU exceeded 90.4%, the mean axial localization error was 2.448 mm, and the average online processing time was 53.35 ms per image. The key performance metrics obtained from the deployed-system validation.are listed in Table 15.

### 4.9. Curvature-Sensitivity Analysis and Applicability Constraint

A first-order analytical curvature-sensitivity analysis was conducted using the cylindrical cross-section and error bounds defined in Section 3.6. The analysis evaluated local curvature radii of 1500–4000 mm, physical localization-region widths of 100–200 mm, and viewing angles of 15–30°. The representative acquisition working distance was 500–600 mm, but it was not an explicit variable in the adopted first-order analytical error bound. For each parameter combination, the maximum sagitta, spatially averaged axial error, maximum axial-error bound, and estimated projected bounding-box IoU loss were calculated.

The maximum sagitta was calculated as $s_{max}=\rho -\sqrt{\rho^{2}-{\left(\frac{W}{2}\right)}^{2}}$. The mean axial error was estimated by spatially averaging the circular-segment sagitta across the localization region and multiplying it by tanθ, whereas the maximum axial-error bound was calculated as $e_{max}=s_{max}tan\theta$. The IoU loss was estimated for a W-wide rectangular region subjected to an axial displacement of *e*_max_. Working distance specifies the representative acquisition geometry; it does not explicitly enter the adopted first-order error bound.

**Figure 13:**
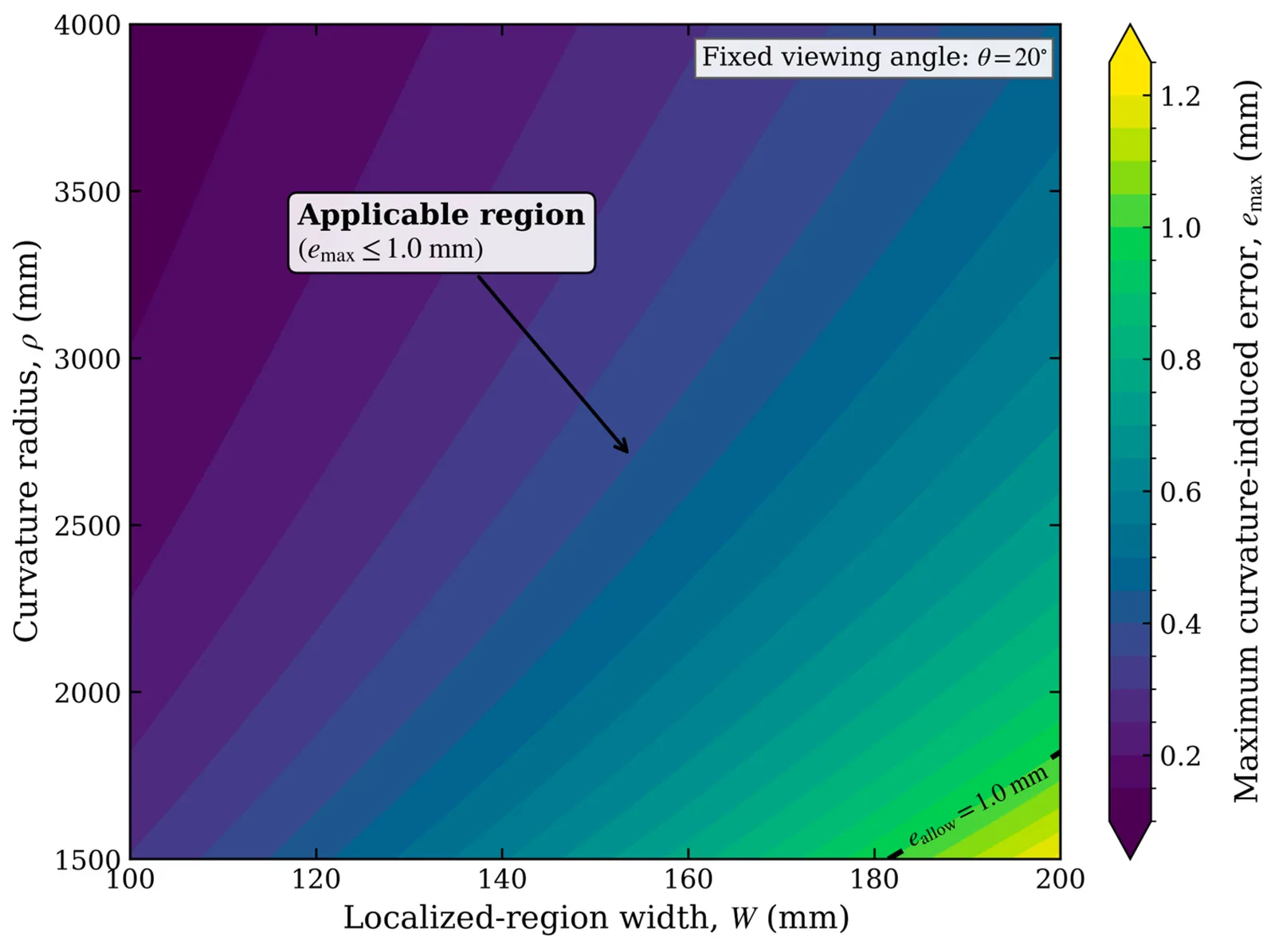
Curvature-induced maximum axial localization error as a function of local curvature radius and inspection-region width.

As shown in Table 16 and Figure 13, the curvature-induced axial localization error increased as the curvature radius decreased, the inspection-region width increased, or the viewing direction became more oblique. Within the recommended analytically assessed operating domain of ρ ≥ 1500 mm, W ≤ 150 mm, and θ ≤ 20°, the estimated maximum additional axial error did not exceed 0.683 mm, remaining below the allowable limit of 1.0 mm. Parameter combinations outside this domain require cylindrical or general curved-surface ray-intersection modeling.

## 5. Discussion

### 5.1. Practical Effect of Cross-Validated Detector-Center Correction

A six-fold leave-one-image-out procedure was used to prevent an evaluated image from contributing to its own center-shift parameters. The cross-validated ablation results show that center-shift correction only provided the best overall performance, reducing the MAE from 2.179 to 0.641 mm and increasing the projected physical-plane IoU from 63.45% to 87.12%. Size correction alone did not reduce the MAE, whereas combined center-and-size correction produced an MAE of 0.664 mm and an IoU of 86.66%. Therefore, center-shift correction only was retained.

Because the six images participated in different cross-validation folds, the result represents internal cross-validated generalization rather than performance on a completely external metrology dataset. Additional independently measured multi-specimen data remain necessary before the correction can be treated as universally transferable.

### 5.2. Benchmarking Against Conventional and Published Vision-Localization Methods

The quantitative comparison demonstrates that the physical-localization performance is influenced not only by detector accuracy but also by the image-to-surface conversion strategy. Because all automatic methods used the same detector-generated bounding boxes, the differences in localization performance primarily arose from their geometric mapping formulations.

Local pixel-to-millimeter scaling assumes an approximately uniform image scale and does not explicitly represent camera pose, perspective projection, or lens distortion. Planar homography models the projective relationship between the image and the inspected surface. The conventional calibrated camera-mapping baseline further incorporates intrinsic, extrinsic, and distortion parameters, but it directly maps the detector-generated boxes without correcting their systematic boundary bias. In contrast, the correction was applied after original-coordinate restoration, lens-distortion correction, and calibrated ray-to-surface mapping.

Across the six held-out folds, the proposed framework achieved a mean absolute axial localization error of 0.641 mm. This corresponds to a reduction of 97.91% relative to local pixel-to-millimeter scaling and 70.58% relative to conventional calibrated camera mapping. The proposed method achieved accuracy comparable to planar homography mapping, which produced an MAE of 0.616 mm; the corresponding RMSE values were 0.809 mm and 0.825 mm, respectively, and both methods achieved 98.81% of axial errors within 3 mm. Therefore, the proposed method should not be claimed to outperform planar homography in MAE, but it substantially improved the conventional calibrated mapping result by compensating for detector-output center bias.

### 5.3. Relationship to Image-Domain Aircraft Corrosion Detection

Most image-domain corrosion inspection methods output corrosion categories, bounding boxes, or masks in pixel coordinates. Such outputs are useful for recognition but cannot directly provide metric surface coordinates for maintenance execution. The proposed method extends image-domain detection to physical-surface localization by coupling the detector-generated box with camera calibration and surface-plane projection. This makes the output more suitable for targeted maintenance because the corrosion region is represented in millimeters on the fuselage surface rather than only in pixels.

### 5.4. Limitations and Future Work

Although the revised experiments include an independent 214-image detector test and a separate ten-image engineering deployment evaluation, the detailed six-image physical-localization result remains a leave-one-image-out cross-validated assessment rather than an independent external metrology evaluation.

#### 5.4.1. Influence of Local Surface Curvature

The current camera-mapping model represents the inspected fuselage region using a local tangent plane. When the actual fuselage surface deviates from this plane, the camera ray intersects the assumed plane and the actual curved surface at different spatial positions, thereby introducing a model-form localization error. This error increases as the local curvature radius decreases, the physical width of the inspected region increases, or the viewing direction becomes more oblique.

The first-order analytical analysis in Section 4.9 indicates that the recommended operating domain of the tangent-plane mapping model is defined by ρ ≥ 1500 mm, W ≤ 150 mm, and θ ≤ 20°. Within this domain, the estimated maximum curvature-induced additional axial error did not exceed 0.683 mm. The working-distance range of 500–600 mm describes the representative acquisition configuration but does not explicitly enter the adopted first-order bound. This domain is an analytically assessed recommendation rather than a universally validated curvature limit.

Future work will replace the single-plane representation with piecewise tangent-plane, cylindrical-surface, or general parametric-surface models. A practical implementation route is to estimate the local surface normal and curvature from aircraft CAD geometry, calibrated structural reference points, or depth observations, and then compute the intersection between each camera ray and the reconstructed curved surface rather than with a fixed plane.

In a future adaptive implementation, the local curvature radius and inspection-region width will first be estimated from aircraft CAD geometry, structural reference points, or depth observations. The curvature-induced error will then be predicted using the applicability model established in Section 3.6. If the predicted error remains below e_allow_, the local tangent-plane model will be retained. Otherwise, the system will automatically switch to cylindrical or mesh-based ray–surface intersection. For general curved surfaces, the localized corrosion contour should be represented using surface or geodesic coordinates rather than an axis-aligned rectangle defined on a tangent plane.

#### 5.4.2. Influence of Uneven Illumination and Surface Reflection

Aircraft fuselage surfaces may exhibit strong reflection, shadowing, local overexposure, and non-uniform illumination. These photometric variations do not directly alter the camera geometric model, but they affect the apparent corrosion contrast and boundary visibility. Consequently, the detector may produce lower confidence scores, shifted box centers, or expanded and contracted boundaries. Because the physical-localization module uses detector-generated corners as its geometric input, such image-domain deviations are subsequently propagated into millimeter-scale physical-coordinate errors.

In addition, the detector-boundary calibration vector estimated from the validation subset assumes that the statistical distribution of detector boundary bias remains approximately stable. Severe illumination changes may alter this distribution and reduce the transferability of the fixed calibration vector. Directional image-analysis studies suggest that local anomalies need not affect all spatial directions equally. A crack, reflective streak, fastener edge, or local contamination region may corrupt only part of the corrosion boundary. Multidirectional region analysis and consistency checking could therefore be used to identify unreliable boundary sectors before physical-coordinate projection. However, unlike tree-ring structures, aircraft corrosion regions do not generally possess a common radial center or periodic peak pattern. Consequently, pith localization and radial peak counting cannot be transferred directly to the present task. A more appropriate extension would divide each corrosion boundary into multiple oriented sectors, estimate sector-level edge confidence, and reject or down-weight inconsistent sectors before fitting the final corrosion contour or bounding box.

Future work will combine cross-polarized or multi-angle illumination, automatic exposure control, and high-dynamic-range image acquisition to suppress specular reflection and shadowing. At the algorithmic level, illumination normalization, illumination-aware data augmentation, and domain-generalization training will be investigated. The fold-specific center-shift correction assumes that the statistical distribution of detector-center bias remains approximately stable across the evaluated acquisition conditions. Severe illumination changes may alter this distribution and reduce the transferability of the estimated correction.

A practical reflection-handling pipeline will first detect overexposed or strongly polarized regions using intensity saturation, local contrast, and polarization-response indicators. Boundary sectors overlapping unreliable reflective regions will then be assigned lower confidence during contour or bounding-box estimation. Images acquired under multiple illumination directions may be registered and fused so that corrosion boundaries obscured in one image can be recovered from another. The detector-boundary calibration may further be conditioned on reflection coverage, mean intensity, local contrast, and detector confidence rather than using one global correction vector for all photometric conditions.

#### 5.4.3. Influence of Viewpoint and Camera-Pose Variation

The current physical-coordinate mapping relies on intrinsic and extrinsic parameters obtained under a calibrated camera configuration. When the camera position or orientation changes beyond the calibrated range, the previously estimated rotation matrix and translation vector may no longer accurately describe the relationship between the camera and the inspected surface. Such extrinsic-parameter mismatch produces systematic coordinate errors even when corrosion detection remains correct. Oblique viewing also increases perspective compression and makes small pixel deviations correspond to larger physical-coordinate changes in some image regions. In-plane image rotation augmentation can improve detector robustness to changes in image orientation, but it does not reproduce the full geometric effects of an out-of-plane camera-pose change. A true viewpoint change modifies perspective projection, apparent defect scale, occlusion, surface reflection, and the relationship between image coordinates and physical-surface coordinates. Moreover, arbitrary-angle rotation may introduce interpolation artifacts, cropped image boundaries, or artificial padding. Rotation augmentation should therefore be regarded as a detector-training strategy rather than a substitute for camera-pose calibration or geometric remapping.

The present robotic platform reduces this problem by controlling the camera pose, but positioning errors, manipulator compliance, and repeated installation may still introduce pose variation. Therefore, the reported localization accuracy should not be generalized to arbitrary camera poses without recalibration or pose verification.

Future work will introduce an online pose-validity check based on calibration-point reprojection error. When the reprojection error exceeds a predefined threshold, the camera extrinsic parameters will be re-estimated using temporary fiducial markers, fuselage structural features, or automatically detected reference points. Hand–eye calibration between the robotic manipulator and camera, together with a multi-pose calibration library, will also be investigated to support localization under a wider range of camera orientations and working distances.

#### 5.4.4. Influence of Coexisting Corrosion and Surface-Damage Types

The present localization evaluation mainly focuses on individually identifiable pitting-corrosion regions. In practical aircraft inspection, several forms of surface damage may coexist within the same field of view, including pitting corrosion, crevice corrosion, galvanic corrosion, coating peeling, scratches, fastener-related damage, and crack-like features. These damage types may overlap spatially or exhibit similar low-contrast and irregular appearances.

Coexisting damage can affect the proposed framework at both the perception and physical-mapping stages. At the perception stage, overlapping features may cause class confusion, missed detections, or merged bounding boxes. Conventional non-maximum suppression may remove one valid prediction when two damage regions overlap strongly. At the physical-mapping stage, a merged detector box may be projected as one large physical region, thereby overestimating the area assigned to an individual defect. In addition, the current fold-specific center-shift correction may not remain appropriate because different damage types can exhibit different boundary contrast, morphology, and detector-box bias.

Future work will replace the current single-class rectangular-box representation with a hierarchical multi-damage perception strategy. A first-stage detector will identify candidate damaged regions, followed by a multi-label instance-segmentation module that separates overlapping damage types and estimates their individual contours. Class-aware or soft non-maximum suppression will be used to preserve valid overlapping instances. Each damage type will be assigned a class-specific or morphology-conditioned detector-center correction model before physical-coordinate projection.

For physical representation, each segmented damage contour will be projected independently onto the calibrated surface rather than enclosing all coexisting damage within one rectangular box. The system will report the damage class, physical contour, affected surface area, confidence, and uncertainty for each instance. When damage classes cannot be separated reliably, the region will be flagged for manual review rather than being reported as a high-confidence metric localization result.

#### 5.4.5. Potential and Cost of Multimodal Fusion

Multimodal fusion offers a potential route for reducing ambiguities that cannot be resolved from RGB appearance alone. Visible images provide texture, color, and structural context, whereas near-infrared, thermal, depth, or other sensing modalities may provide complementary responses under weak contrast, coating variation, or non-uniform illumination. Recent multimodal parallel-attention architectures demonstrate that modality-specific feature extraction followed by attention-based fusion can improve the representation of visually ambiguous targets [39].

However, multimodal fusion is not included in the current framework. Additional sensors would require spatial and temporal registration, radiometric calibration, synchronization, and modality-specific quality control. Registration errors between modalities could themselves propagate into the physical-coordinate localization result, while parallel feature extraction would increase computational and deployment complexity. Therefore, the current study uses a monocular RGB camera to preserve a lightweight calibrated measurement chain.

A concrete future implementation route would combine RGB corrosion texture with infrared thermal contrast or depth-derived surface geometry. Modality-specific encoders could first extract RGB, thermal, and geometric features, followed by confidence-weighted cross-modal attention. The fused perception result would then be projected through a shared surface-coordinate model. An uncertainty gate would suppress a modality when its registration residual or signal quality exceeded a predefined threshold.

#### 5.4.6. Transferability of Detector-Center Correction

The fold-specific center-shift correction assumes that the statistical distribution of detector-center bias remains approximately stable across the evaluated acquisition conditions. Severe illumination changes may alter this distribution and reduce the transferability of the estimated correction. Therefore, the current correction parameters should not be interpreted as universal constants applicable to all corrosion-inspection systems. Future work will investigate scale-, confidence-, morphology-, and illumination-conditioned detector-center correction instead of using a single global center shift. A probabilistic center-shift model may be used to predict both the required center correction and its uncertainty interval for each corrosion instance. Pixel-level corrosion segmentation will also be evaluated as an alternative to rectangular detection boxes.

#### 5.4.7. Physical-Reference Uncertainty and Experimental Scope

The physical-coordinate references were established through manual corrosion-boundary marking, coordinate-origin transfer, and engineering measurement. These references therefore contain finite uncertainty and should not be interpreted as exact ground truth. In the revised experiment, repeated measurements from two independent operators were used to quantify boundary-marking and reading repeatability. Measurement-tape resolution and repeated coordinate-system establishment were additionally included in the uncertainty budget.

The combined standard uncertainty of the manually established physical-reference coordinates was 0.187 mm, corresponding to an expanded uncertainty of 0.374 mm at k = 2. After propagation through 10,000 Monte Carlo trials, the mean absolute axial error was 0.656 mm, with a 95% interval of 0.618–0.693 mm. Compared with the directly observed MAE of 0.641 mm, the reference uncertainty was smaller than, but not negligible relative to, the reported localization-error scale.

Nevertheless, manual identification of irregular corrosion boundaries remains partly subjective, particularly under weak contrast and non-uniform illumination. Future experiments will use higher-precision coordinate-measurement equipment, structured-light scanning, or surface photogrammetry to provide independently traceable reference geometry and further reduce boundary-definition uncertainty.

## 6. Conclusions

This study presented a camera-calibrated vision measurement framework with Cross-Validated Detector-Center Correction for physical-surface localization of aircraft fuselage corrosion. PWDE-YOLOv8n was adopted as the corrosion perception module to provide bounding boxes, while the main focus was the conversion of image-space detections into metric surface coordinates. In each fold, the physical-plane center shift was estimated exclusively from the other five images and applied unchanged to both mapped corners of the held-out image. By combining inverse coordinate restoration, distortion compensation, camera calibration, and planar geometric projection, the detected corrosion regions were mapped from pixel coordinates to fuselage-surface coordinates. After physical-specimen- and acquisition-session-level dataset repartitioning and detector retraining, the framework was evaluated using six-fold leave-one-image-out cross-validation on six images containing 21 corrosion boxes with manually measured physical-surface references. The revised evaluation achieved a mean absolute axial localization error of 0.641 mm (95% CI: 0.571–0.708 mm), an RMSE of 0.809 mm, a maximum axial error of 3.262 mm, and an average projected bounding-box IoU of 87.12%. The expanded uncertainty of the manually established reference coordinates was 0.374 mm at k = 2, and Monte Carlo propagation produced a 95% measurement-uncertainty interval of 0.618–0.693 mm for the mean absolute axial localization error. These results support the feasibility of the proposed detection-to-surface localization framework under the current calibrated and locally planar operating conditions. The six-image benchmark averaged 41.05 ms/image, whereas the separate ten-image engineering deployment evaluation averaged 53.35 ms/image under online acquisition conditions; both results support near-real-time inspection. The detector architecture itself is not the main contribution of this work; rather, the contribution lies in the calibrated coordinate conversion and boundary-uncertainty-aware evaluation of detector-generated corrosion boxes in physical-surface coordinates.

The proposed method substantially outperformed local pixel-to-millimeter scaling and conventional calibrated camera mapping, while its MAE was comparable to that of planar homography mapping rather than lower than it. The comparison with the conventional calibrated baseline demonstrates the additional contribution of Cross-Validated Detector-Center Correction beyond standard image-to-surface projection.

The present study still has several limitations. The localization performance may be affected by severe illumination non-uniformity, large viewpoint changes beyond the calibrated operating range, and the planar approximation used for locally curved fuselage surfaces. Future work will extend the current framework through six concrete technical routes:

(1) Replacing the single-plane model with piecewise tangent, cylindrical, or parametric curved-surface ray-intersection models;
(2) Introducing online extrinsic-parameter verification and re-estimation for large camera-pose changes;
(3) Integrating polarized illumination, exposure control, reflection-region confidence estimation, and illumination-aware domain generalization;
(4) Developing multi-label instance segmentation, class-aware post-processing, and damage-specific correction for coexisting corrosion and surface-damage types;
(5) Developing condition-adaptive detector-center correction and pixel-level corrosion segmentation;
(6) Constructing higher-accuracy physical references using repeated multi-operator measurement or three-dimensional surface scanning.

## Figures and Tables

**Table 1:** Role of the dataset sources in the proposed localization framework.

Dataset Category	Number of Images	Number of Annotations	Role in the Present Localization Framework
Laboratory-prepared simulated corrosion samples	1549	4511	Provide sufficient image-domain corrosion boxes for detector-based coordinate mapping
Real corrosion, including fuselage-related experimental samples and screened real-scene images	594	1430	Support evaluation under more practical corrosion appearances, backgrounds, and illumination conditions
Total	2143	5941	Used for image-domain corrosion extraction and subsequent physical-surface localization evaluation

**Table 2:** Group-level dataset partitioning used in the revised experiments.

Dataset Subset	Specimen/Session or Source Groups	Original Images	Corrosion Annotations	Experimental Role
Training set	18	1500	4159	Detector parameter learning
Validation set	6	429	1189	Model selection and threshold determination
Independent detector test set	6	214	593	Final image-domain detector evaluation; not used for model selection or threshold adjustment
Total	30	2143	5941	-

**Table 3:** Distinction between image-formation edge spreading and detector-output box bias.

Characteristic	Image-Formation Edge Spreading	Detector-Output Box Bias
Processing stage	Optical imaging and sensor sampling	Detection, regression, and post-processing
Mathematical descriptor	PSF parameter σ, edge-spread width w_10–90_	Center, width, height, or four-boundary residuals
Primary causes	Defocus, diffraction, motion, pixel integration, noise	Image blur, resizing, downsampling, feature aggregation, box regression, NMS, annotation ambiguity
Direct consequence	Finite-width image-intensity transition	Translation, expansion, or contraction of the predicted box
Treatment in this study	Theoretical upstream explanation	Fold-specific cross-validated physical-plane center-shift correction

**Table 4:** Key hardware parameters.

Hardware Device	Specification	Parameter
HBVCAM-4K2353V22 Camera	Resolution (Pixel)	2592 × 1944
	Focal Length (mm)	4
	Power Supply (V)	DC-12
BT-23C0814MP5 Lens	Focal Length (mm)	8
	Interface Type	C-Mount
	Image-Plane Size (inch)	1/1.8″
MV-RL51X25W-F Light Source	Light-Source Color	White
	Emitting (mm)	Φ50 × Φ20
	Dimensions(mm)	Φ50 × Φ20 × Φ23
Reconfigurable Compliant Manipulator for Maintenance	Effective Length (cm)	112
	Rated Torque (N·m)	2.5

**Table 5:** Uncertainty budget for the manually established physical-coordinate references.

Uncertainty Source	Evaluation Method	Distribution/Model	Standard Uncertainty/mm
Repeated boundary marking and coordinate reading	Two operators × three repetitions	Experimental standard deviation	0.144
Measurement-tape resolution	$d/\sqrt{12}$, d = 0.1 mm	Rectangular	0.029
Coordinate-origin and axis establishment	Repeated complete setup experiments	Experimental standard deviation	0.115
Gauge-block contribution	Calibration certificate, when available	Certificate value	0.001
Combined standard uncertainty	Root sum of squares	-	0.187
Expanded uncertainty, k = 2	$U_{ref}=ku_{ref}=0.374\;mm$	Approximate 95% coverage	0.374

**Table 6:** Sources of detector-output box uncertainty and their treatment in the proposed framework.

Detector-Output Boundary-Uncertainty Source	Possible Effect on Detector-Generated Corrosion Box	Treatment in the Proposed Framework
Convolutional receptive-field aggregation	Boundary smoothing or inclusion of surrounding weak-texture regions	Treat detector-generated boxes as boundary-uncertain perception outputs rather than exact physical boundaries.
Feature-map downsampling and multi-scale fusion	Spatial offset or scale-dependent box broadening	Restore boxes from detector-input coordinates to original-image coordinates before projection
Image resizing to 640 × 640	Coordinate-scale distortion if directly projected	Apply inverse coordinate restoration before distortion correction and camera mapping
Non-maximum suppression and confidence filtering	Selection of a slightly larger or shifted final box	Evaluate the resulting error through surface-plane IoU and axial physical-coordinate error
Manual reference and calibration uncertainty	Additional discrepancy between mapped detector box and measured surface box	Report physical-coordinate errors with explicit acknowledgement of measurement uncertainty

**Table 7:** Center-shift parameters used in the six-fold leave-one-image-out evaluation.

Held-Out Image	Images Used for Estimation	Number of Boxes Used	Equivalent X-Shift (mm)	Equivalent Y-Shift (mm)
1	2–6	15	2.105	1.965
2	1, 3–6	19	2.130	2.000
3	1–2, 4–6	16	2.158	2.100
4	1–3, 5–6	19	2.190	2.120
5	1–4, 6	20	2.160	2.100
6	1–5	16	2.218	2.128

**Table 8:** Ablation study of detector-center and size correction under six-fold leave-one-image-out evaluation.

Method	MAE/mm	RMSE/mm	Maximum/mm	Projected Box IoU/%	Error ≤ 3mm/%
Raw detector boxes	2.179	2.315	5.420	63.45	85.71
Center-shift correction only	0.641	0.809	3.262	87.12	98.81
Size correction only	2.179	2.319	5.267	63.33	88.10
Center-and-size correction	0.664	0.819	3.109	86.66	98.81

**Table 9:** Image-plane coordinate transformation.

Index	Box Center	Width	Height	Upper-Left Corner	Lower-Right Corner	Sample Category
1	(1249.5, 791.5)	177	135	(1161, 724)	(1338, 859)	Mild corrosion
	(1385.0, 585.0)	176	154	(1297, 508)	(1473, 662)	Mild corrosion
	(355.0, 709.5)	198	191	(256, 614)	(454, 805)	Mild corrosion
	(1164.5, 572.5)	191	187	(1069, 479)	(1260, 666)	Mild corrosion
	(1160.5, 572.0)	193	190	(1064, 477)	(1257, 667)	Moderate corrosion
	(1387.5, 586.5)	177	157	(1299, 508)	(1476, 665)	Moderate corrosion
2	(1340.5, 626.0)	263	238	(1209, 507)	(1472, 745)	Moderate corrosion
	(1145.5, 823.0)	253	208	(1019, 719)	(1272, 927)	Moderate corrosion
3	(550.5, 924.0)	175	180	(463, 834)	(638, 1014)	Moderate corrosion
	(350.5, 1074.5)	201	131	(250, 1009)	(451, 1140)	Moderate corrosion
	(742.0, 1074.5)	202	165	(641, 992)	(843, 1157)	Mild corrosion
	(805.5, 820.0)	167	198	(722, 721)	(889, 919)	Mild corrosion
	(742.0, 1073.0)	202	168	(641, 989)	(843, 1157)	Moderate corrosion
4	(694.5, 842.0)	407	390	(491, 647)	(898, 1037)	Moderate corrosion
	(693.0, 846.5)	404	391	(491, 651)	(895, 1042)	Moderate corrosion
5	(718.0, 827.0)	364	360	(536, 647)	(900, 1007)	Moderate corrosion
6	(1104.5, 991.0)	157	128	(1026, 927)	(1183, 1055)	Mild corrosion
	(1334.0, 796.5)	178	149	(1245, 722)	(1423, 871)	Mild corrosion
	(1135.5, 771.5)	155	113	(1058, 715)	(1213, 828)	Mild corrosion
	(1354.0, 1025.0)	156	148	(1276, 951)	(1432, 1099)	Moderate corrosion
	(1355.0, 1025.5)	158	147	(1276, 952)	(1434, 1099)	Mild corrosion

**Table 10:** Cross-validated corrected physical coordinates and axial localization errors for the six-image metrology cohort.

Index	Corrosion ID	Manual Reference Coordinates/mm	Cross-Validated Corrected Coordinates/mm	Absolute Axial Localization Errors/mm
1	1.1	(93.2, 28.5), (80.6, 47.1)	(94.325, 29.905), (81.255, 47.275)	(1.125, 1.405), (0.655, 0.175)
	1.2	(114.3, 41.5), (100.2, 59.9)	(115.155, 42.565), (100.415, 60.335)	(0.855, 1.065), (0.215, 0.435)
	1.3	(103.5, −57.3), (86.1, −38.5)	(104.585, −57.305), (86.325, −38.645)	(1.085, 0.005), (0.225, 0.145)
	1.4	(117.2, 20.1), (100.4, 39.4)	(117.815, 20.945), (99.955, 39.535)	(0.615, 0.845), (0.445, 0.135)
	1.5	(117.4, 20.3), (99.2, 39.1)	(118.005, 20.465), (99.855, 39.245)	(0.605, 0.165), (0.655, 0.145)
	1.6	(114.2, 42.1), (100.5, 59.7)	(115.155, 43.245), (100.125, 60.625)	(0.955, 1.145), (0.375, 0.925)
2	2.1	(115.2, 33.5), (92.1, 59.8)	(115.180, 34.470), (92.350, 60.280)	(0.020, 0.970), (0.250, 0.480)
	2.2	(95.1, 16.4), (75.2, 40.2)	(94.740, 16.060), (74.580, 40.810)	(0.360, 0.340), (0.620, 0.610)
3	3.1	(84.2, −37.1), (67.1, −21.4)	(83.482, −37.950), (66.022, −21.230)	(0.718, 0.850), (1.078, 0.170)
	3.2	(67.2, −58.2), (53.1, −39.2)	(66.502, −58.800), (53.692, −39.570)	(0.698, 0.600), (0.592, 0.370)
	3.3	(64.9, −22.3), (52.5, −2.1)	(68.162, −20.920), (51.982, −1.350)	(3.262, 1.380), (0.518, 0.750)
	3.4	(93.8, −13.4), (75.2, 2.1)	(94.432, −12.820), (75.302, 3.260)	(0.632, 0.580), (0.102, 1.160)
	3.5	(68.3, −20.3), (52.2, −1.7)	(68.462, −20.920), (51.982, −1.350)	(0.162, 0.620), (0.218, 0.350)
4	4.1	(102.1, −34.1), (64.1, 5.6)	(101.430, −35.010), (63.740, 4.070)	(0.670, 0.910), (0.360, 1.530)
	4.2	(100.7, −34.2), (64.1, 3.2)	(101.050, −35.010), (63.250, 3.770)	(0.350, 0.810), (0.850, 0.570)
5	5.1	(102.3, −30.1), (66.5, 4.9)	(101.480, −30.670), (66.710, 4.300)	(0.820, 0.570), (0.210, 0.600)
6	6.1	(75.1, 16.9), (63.1, 32.2)	(74.472, 16.592), (61.942, 31.992)	(0.628, 0.308), (1.158, 0.208)
	6.2	(94.5, 38.2), (80.1, 55.3)	(94.432, 37.932), (79.982, 55.472)	(0.068, 0.268), (0.118, 0.172)
	6.3	(95.3, 18.3), (84.1, 33.9)	(95.052, 19.722), (84.132, 34.852)	(0.248, 1.422), (0.032, 0.952)
	6.4	(72.3, 41.4), (57.2, 57.8)	(72.152, 41.082), (57.592, 56.562)	(0.148, 0.318), (0.392, 1.238)
	6.5	(73.2, 40.2), (59.1, 58.3)	(72.052, 41.082), (57.592, 56.762)	(1.148, 0.882), (1.508, 1.538)

**Table 11:** Corrosion region localization accuracy.

Image Index	Corrected Physical-Plane IoU/%	Maximum Corrected Axial Error/mm
1	86.83	1.405
2	92.57	0.970
3	84.09	3.262
4	92.46	1.530
5	93.97	0.820
6	84.80	1.538
Overall	87.12	3.262

**Table 12:** Processing time of each module in the proposed framework.

Module	Processing Time per Image (ms)
Image Acquisition Module	16.78
Image Enhancement Module	8.17
PWDE-YOLOv8n Corrosion Detection Module	9.58
Corrosion Localization Module	6.52
Total	41.05

**Table 13:** Same-platform comparison of physical-surface localization methods on the six-image cross-validated metrology cohort.

Method	MAE/mm	RMSE/mm	Median/mm	Maximum/mm	Projected Box IoU/%	Error ≤ 3 mm/%	Mapping Time /ms	End-to-End Time/ms
Local pixel-to-millimeter scaling	30.694	35.966	29.117	88.694	0.00	3.57	0.01	34.54
Planar homography mapping	0.616	0.825	0.541	3.750	87.54	98.81	0.03	34.56
Conventional calibrated camera mapping	2.179	2.315	2.135	5.420	63.45	85.71	6.48	41.01
Proposed center-corrected camera mapping	0.641	0.809	0.600	3.262	87.12	98.81	6.52	41.05

**Table 14:** Contextual comparison with representative published aircraft-inspection and monocular vision-localization studies.

Study	Task	Reported Quantitative Result	Comparability
Brandoli et al. [8]	Aircraft fuselage corrosion detection	Precision > 93%	Image-domain detection; no millimeter-scale coordinates
Hou et al. [11]	Robotic multi-hole localization	Combined tracking error within 1.5 mm; direction-specific vertical errors up to 5 mm	Different object and robot-tracking error definition
Yang et al. [35]	Air-rudder surface defect positioning	Maximum absolute error 0.53 mm; maximum uncertainty 0.26 mm	Similar camera-mapping concept but different structure and metric
Proposed method	Aircraft fuselage corrosion surface localization	MAE 0.641 mm; RMSE 0.809 mm; maximum 3.262 mm; IoU 87.12%; 41.05 ms/image	Physical corrosion-box coordinates under the present setup

**Table 15:** Summary of deployed-system validation performance.

Module/Metric	Result
Corrosion perception module	PWDE-YOLOv8n
Online localization-test images	10
Input size	640 × 640
Detector inference time	9.58 ms
Average surface-plane IoU	>90.4%
Mean axial localization error	2.448 mm
Average online processing time	53.35 ms

**Table 16:** Analytical estimates of curvature-induced localization errors under representative operating conditions.

Curvature Radius ρ/mm	Region Width W/mm	View Angle/°	Maximum Sagitta/mm	Mean Axial Error/mm	Maximum Axial Error/mm	IoU Loss/Percentage Points
4000	100	15	0.313	0.028	0.084	0.17
2500	150	20	1.125	0.137	0.410	0.54
1500	150	20	1.876	0.228	0.683	0.91
2500	200	30	2.001	0.385	1.155	1.15
1500	200	30	3.337	0.642	1.927	1.91

## Data Availability

The data presented in this study are available from the corresponding author upon reasonable request, subject to restrictions related to ongoing research and aircraft-structure inspection data.
